# Diastereoselective functionalisation of benzo-annulated bicyclic sultams: Application for the synthesis of *cis*-2,4-diarylpyrrolidines

**DOI:** 10.3762/bjoc.5.69

**Published:** 2009-11-25

**Authors:** Susan Kelleher, Pierre-Yves Quesne, Paul Evans

**Affiliations:** 1 Centre for Synthesis and Chemical Biology, School of Chemistry and Chemical Biology, University College Dublin, Dublin 4, Ireland

**Keywords:** cyclic sulfonamides, diastereoselective alkene functionalisation, double reduction, Pd-mediated cross coupling

## Abstract

The *cis*-dibromination of unsaturated bicyclic bridgehead sultams **5a** and **5b**, and experiments designed to understand the *cis*-stereochemical outcome of these reactions, are described. In the case of **5b**, a novel solvent dependent carbocation rearrangement occurs with the formation of **18b**. *cis*-Dibromides **13a** and **13b** undergo regioselective dehydrobromination, and the participation of the resultant vinyl bromide **24a** in lithiation and Pd-coupling chemistry is described. In the case of the latter, hydrogenation of the styryl products afforded a single diastereoisomer. These compounds were then studied under dissolved metal reduction conditions, in which the cleavage of both N–S and C–S bonds takes place to afford *cis*-2,4-diaryl-substituted pyrrolidines **35–37**.

## Introduction

**Scheme 1:**
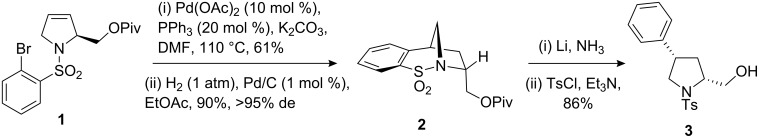
The diastereoselective intramolecular Heck-hydrogenation and double reduction sequence as a means of accessing *cis*-disubstituted pyrrolidine **3**.

Substituted pyrrolidine ring systems represent a common structural motif in a range of biologically active compounds, including pharmaceutical agents and natural products. In relation to these general targets, we have recently developed a method that enables the construction of aryl-substituted pyrrolidines, featuring the double reduction of cyclic aromatic sulfonamides [1–4]. As illustrated in Scheme 1, a Heck (Heck–Mizoroki) cyclisation [5–9] was employed to form the cyclic sulfonamide. Subsequently, it was shown that high stereofacial bias was achieved on hydrogenation, generating **2** as a single diastereoisomer. Treatment under dissolved metal reduction conditions afforded the *cis*-disubstituted pyrrolidine. In this sequence, the sulfonyl moiety not only serves as an amino protecting group but also facilitates the diastereoselective intramolecular carbon–carbon bond formation. In this present study we demonstrate that this general concept may be extended enabling the diastereoselective preparation of 2,4-diaryl-substituted pyrrolidines from more readily available, albeit racemic, substrates.

## Results and Discussion

**Scheme 2:**
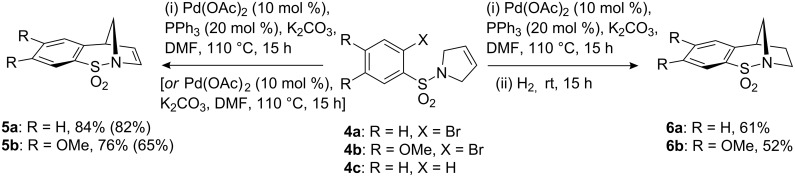
The synthesis of **5a** and **5b** by an intramolecular Heck cyclisation reaction.

Bicyclic aromatic cyclic sulfonamides **5a** and **5b** were formed according to the 3-step sequence previously described [1–3,10]. Originally, inclusion of triphenylphosphine was employed, however, optimisation of this reaction with the exclusion of PPh_3_ gave products **5a** and **5b** in 82 and 65% yields, respectively, which are comparable with the phosphine-based method (**5a**: 84% and **5b**: 76% with PPh_3_) [11]. Standard alkenyl hydrogenation (not shown) then gave the saturated bicycles **6a** and **6b** which were used as substrates for the N–S and C–S bond cleavage forming arylsubstituted pyrrolidines [1–3]. In relation to this sequence attempts to achieve a reductive Heck cyclisation employing ammonium formate [12], gave only the product of bromine-hydrogen exchange **4c** (where X = H). Therefore, a one-pot method was developed based on recent reports which demonstrate that the residual palladium catalyst in Heck reactions may effectively mediate the addition of hydrogen to the newly substituted alkene [13–15]. Thus, following complete Heck cyclisation, as judged by TLC analysis, the reaction was simply stirred under a hydrogen atmosphere in order to afford the corresponding saturated bicycles **6a** and **6b** in yields of 61 and 52% respectively. In terms of efficiency this one-pot process is competitive compared with those from the original two-step, two-pot process (Scheme 2).

**Scheme 3:**
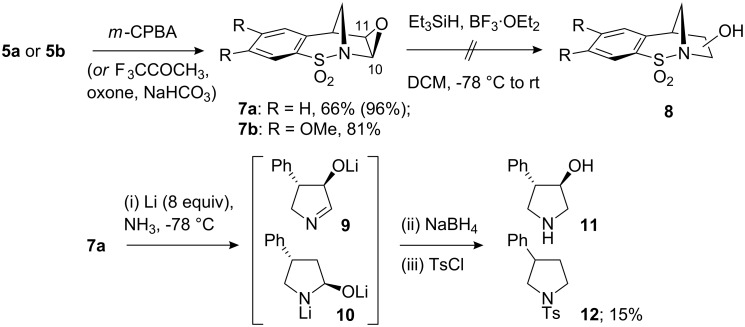
Diastereoselective epoxidation of **5a** and **5b**.

**Figure 1:**
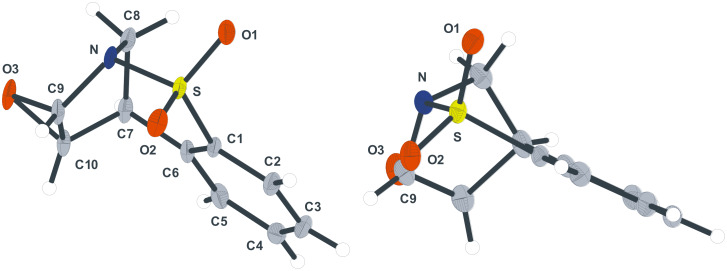
X-ray structure of **7a**; including a view along the S=O···C–O axis (Diamond representations) [O2···C9: 2.850(2) Å].

Functionalisation of the *N*-sulfonyl enamines **5a** and **5b** was subsequently studied with the ultimate aim of introducing substituents, in a stereoselective fashion, to the masked pyrrolidine ring. Thus, epoxidation of **5a** and **5b** (Scheme 3) using *m*-CPBA proceeded smoothly for both substrates and as hoped, occurred under complete stereocontrol (de >95%). The stereochemical outcome was confirmed by single-crystal X-ray crystallography (Figure 1, and see crystallographic data). The use of in situ generated trifluoromethyl-methyldioxirane was also investigated and in the case of **5a**, very efficient formation of **7a** was observed [16–17].

Unfortunately, however, under a variety of conditions it proved impossible to elaborate these species. For example, reductive ring-opening using triethylsilane, conditions previously reported for *N*-sulfonylamino-α,β-epoxides [18–19], led only to recovery of starting material and not the hoped for alcohol **8**. A similar outcome was observed under both Sakurai-type conditions and treatment with potassium cyanide. It seems probable that these failures reflect a combination of the inability of the *N*-lone pair to stabilise the developing α-carbocation (the resultant *N*-sulfonyl iminium ion would be anti-Bredt) and in addition the lone pair is perpendicular to the p-orbital resulting from C–O bond cleavage. Furthermore, it also appears that the aromatic ring, based on the fold of the molecule, represents a steric barrier blocking the approach to the C–O σ* orbital. Since the bicyclic structure was implicated in the lack of epoxide reactivity, double reduction of **7a** was then considered. It was hoped that following N–S bond cleavage, the now electron rich amino species would form an imine **9** that would then undergo further reduction to afford **11**. However, in the event the only isolable product was **12**. Although the yield for this process was low, the isolated product indicated that C11 underwent reduction, presumably prior to N–S bond cleavage.

**Scheme 4:**
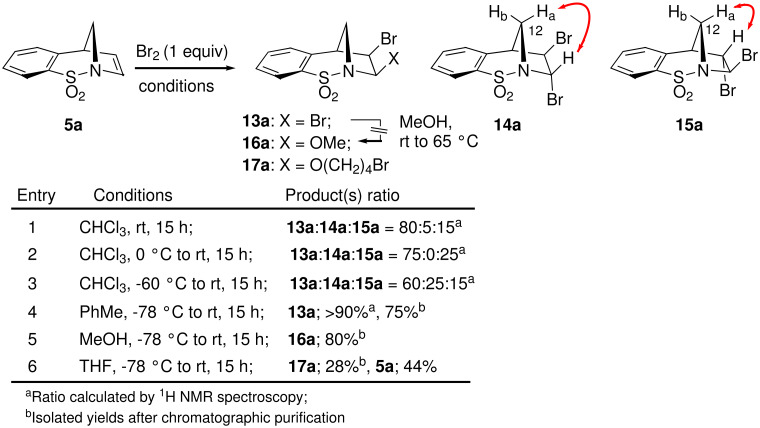
*cis*-Selective dibromination of **5a** (NOE indicated by arrows).

Based on a recent report from Paquette [20], the dibromination of **5a** and **5b** was subsequently considered. We felt that bromination of the double bond would lead to further possibilities for subsequent functionalisation. This report indicated that the treatment of **5a** with neat bromine gave the *cis*-1,2-dibromide **13a** in quantitative yield. It was also reported that under more standard brominating conditions mixtures of diastereoisomeric 1,2-dibromides were formed. In our hands broadly similar results were encountered using chloroform or dichloromethane as reaction solvents (e.g. Entries 1–3). Furthermore, separation of the different diastereoisomers by column chromatography proved difficult. The unusual stereochemical outcome of this bromination process was carefully studied by a combination of NOE analysis, using the diagnostic 12a-CH_2_ signal, and X-ray crystallography which helped to substantiate the structures of the minor 1,2-*trans*-diastereoisomers **14a** and **15a** (see crystallographic data). Optimum conditions, forming **13a** as the major diastereomer, were ultimately found (Scheme 4). Thus, addition of bromine to the compound in toluene at low temperature gave **13a** in 75% isolated yield and high diastereoselectivity as observed by ^1^H NMR spectroscopy of the crude reaction mixture (Entry 4).

**Figure 2:**
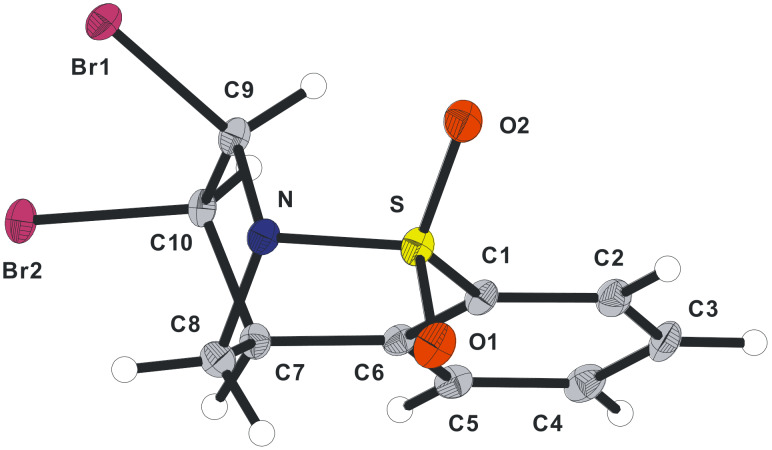
X-ray crystal structure of **13a** (Diamond representation).

In an attempt to further understand the mechanism of this bromination reaction, a bromo-methanolysis reaction was carried out (Entry 5). Interestingly, regio- and diastereoselective formation of the *cis*-**16a** was observed in good yield. When **13a** was taken up in methanol no conversion into **16a**, via an S_N_1-type process, was observed. The use of THF as solvent led to the formation of compound **17a**, albeit in low yield, in which a molecule of the solvent has been incorporated (Entry 6). For X-ray crystal structure of **13a** see Figure 2.

**Scheme 5:**
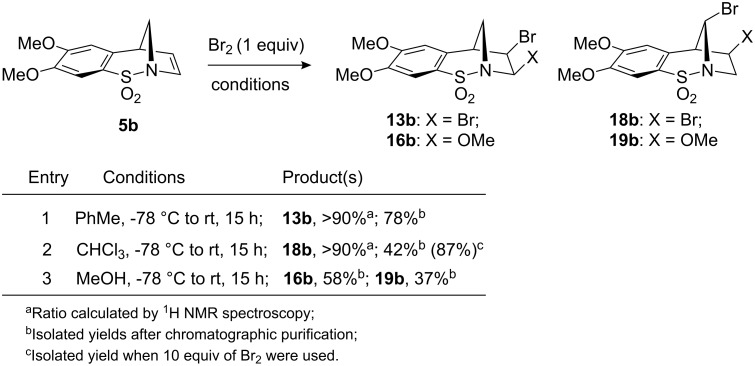
Dibromination studies of **5b**.

The analogous functionalisation of alkene **5b** was then considered. In toluene (Scheme 5, Entry 1), the optimal solvent for the *cis*-dibromination of **5b**, as hoped, selective formation of **13b** was observed (>90% by ^1^H NMR spectroscopy of the crude mixture). It should be noted that whilst the selectivity of this reaction is good, it did prove difficult to perform in a reproducible manner based on the limited solubility of **5b** in toluene and competing benzyl bromide formation in some instances.

**Figure 3:**
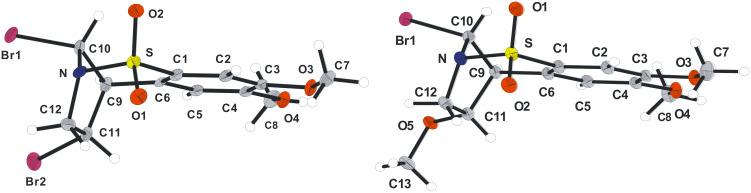
X-ray crystal structures of **18b** and **19b** (Diamond representations).

When the same reaction was conducted in chloroform, the formation of **18b** as the major product (42% yield) was observed (Entry 2). The identity of this compound was determined by single crystal X-ray crystallography (Figure 3). Strikingly, none of this rearranged type of product was observed for **5a** under identical conditions. The yield for this process was increased to 87% when an excess of Br_2_ (10 equiv) was employed. The bromo-methanolysis reaction, performed as described above, gave a mixture of compounds **16b** and **19b** in 58% and 37% yields, respectively (Entry 3). The structure of **19b** was also confirmed by X-ray crystallography (see Figure 3 and crystallographic data).

**Scheme 6:**
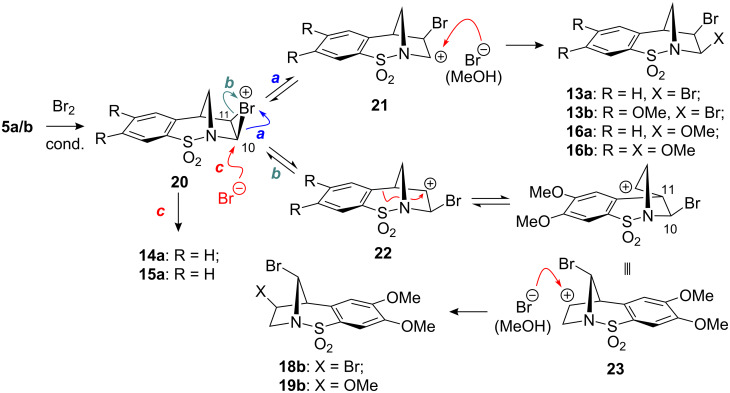
Possible explanation for the products formed in the dibromination of **5a** and **5b**.

Based on the results obtained we propose a mechanistic explanation illustrated in Scheme 6 to account for the products formed. It seems reasonable to speculate that the *cis*-1,2-dibromide formation results from the slow reaction of the bromonium ion **20** with bromide due to the blocking of the lower face by the aromatic ring (route c). Indeed, the epoxide **7a** (Figure 1) may be considered a bromonium ion model and inspection of this representation demonstrates the pronounced fold of this structure. Consequently, since the S_N_2 process is retarded the bromide may then intercept either carbocation **21**, or **22** in an S_N_1 sense, from the less hindered, top face, forming **13a** and **13b**. Using methanol as the solvent the *cis*-1,2-difunctionalised compounds **16a** and **16b** were formed regioselectively (none of the products resulting from methanolysis of **22** were detected). The regiochemical outcome of this process suggests that either the carbocation adjacent to nitrogen is energetically more stable, or that it is more reactive under these conditions. In relation to the *N*-sulfonyl group and its ability to stabilise an α-carbocation, *N*-sulfonyl iminium ions have been implicated in many instances [18–19,21]. However, in this particular case, based on the conformation of the rigid bicyclic molecule, this mode of stabilisation is not feasible. An alternative mechanism of stabilisation has been suggested in which one of the sulfonyl oxygen atoms may act as a Lewis base and in doing so stabilise the positive charge [22–23]. Inspection of the X-ray structure obtained for compounds **7a** and **7b** (not shown) indicates that this stabilisation mechanism may be possible based on the distance between the carbon atom of the epoxide and its closest sulfonyl oxygen atom (see Figure 1).

In the case of carbocation **22**, the benzylic bond appears to be aligned with the empty p-orbital, consequently, when the aromatic ring is substituted with the +M methoxy substituents the results obtained indicate that a Wagner–Meerwein-type rearrangement occurs to generate carbocation **23**. Depending on the conditions this species is then intercepted, again diastereoselectively, by bromide or methanol, affording **18b** and **19b**, respectively. In the case of **5b** in chloroform and methanol this carbocation rearrangement takes place faster than any nucleophilic interception of species **21** and **22**. (A hydride shift from the bridging CH_2_ to carbocation **21** was also considered; however, based on the regioselective formation of compound **19b** this mechanism was discounted.) The remarkable contrasting behaviour for compound **5b** in chloroform and toluene appears to be explained based on the polarity of the respective solvents. Toluene with a low dielectric constant favours formation of **13b** whereas the more polar chloroform facilitates the rearrangement chemistry via carbocation **22**. This type of terpene-like carbocation rearrangement has been reported for molecules which might be considered related to our [2.2.1]-bicyclic systems and has been termed a molecular somersault [24].

**Scheme 7:**
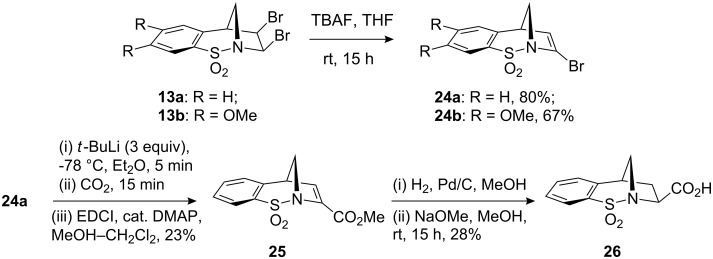
Lithiation–CO_2_ quench approach for the synthesis of **26** from vinyl bromide **24a**.

**Figure 4:**
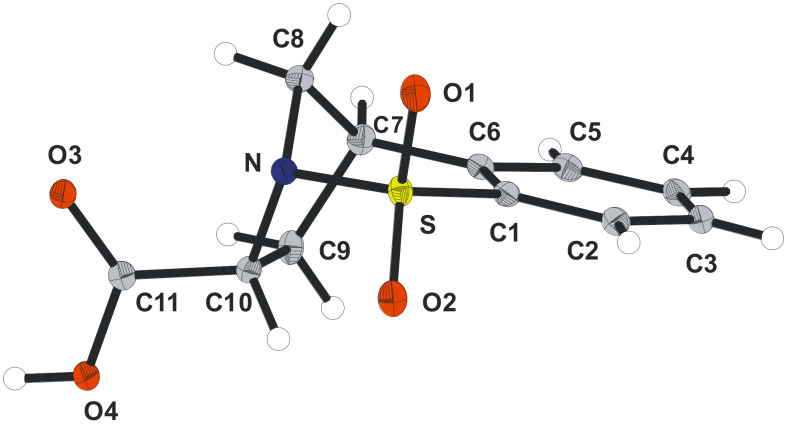
X-ray structure of **26** (Diamond representation).

**Scheme 8:**
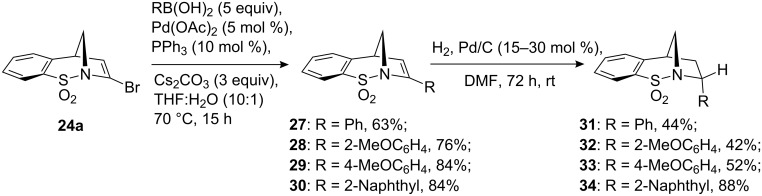
Suzuki–Miyaura cross-coupling of **24a** and the diastereoselective hydrogenation of the resultant styrene adducts.

Regioselective elimination of the *cis*-dibromides **13a** and **13b** with TBAF, according to Paquette’s procedure [20], gave the vinyl bromides **24a** and **24b** in good yields, respectively (Scheme 7). Vinyl bromide **24a** was then subjected to lithiation with *t*-BuLi, followed by a CO_2_ quench. The desired carboxylic acid recovered from this reaction was only formed in low yields possibly due to issues with competing directed *ortho*-lithiation [25]. Notwithstanding, this acid was transformed into the corresponding methyl ester **25** using a Steglich-type esterification. Alkenyl reduction of the α,β-unsaturated ester under standard conditions gave the product of hydrogenation as an undetermined 3:1 mixture of diastereoisomers. The likely explanation for this mixture of products, based on the high levels of diastereoselectivity observed in the similar examples discussed herein, is that the initially formed product begins to undergo epimerisation in order to place the larger (CO_2_Me) substituent on the least hindered face. Consequently, the complete epimerisation of this material was investigated using NaOMe (1 M) based on a literature report [26]. Pleasingly, the hoped for process did indeed lead to the formation of one diastereomer of carboxylic acid **26**, which was formed from the methyl ester on hydrolysis following work-up (see Figure 4 and crystallographic data). Although the yields described during this sequence are low at this stage, the successful epimerisation observed demonstrates that this approach represents a stereo-complementary method to those described in Scheme 1 and Scheme 8.

With the lithiation chemistry described above proving inefficient, palladium chemistry was subsequently considered. The vinyl bromide **24a** was reported to participate in Sonagashira alkynylation chemistry; however, yields of the resultant enyne were low [20]. Therefore, we decided to investigate Suzuki–Miyaura cross-coupling reaction as a means of incorporating alternative structural features into our masked pyrrolidine ring. Under standard conditions, the reactivity of a series of commercially available aryl and alkenyl boronic acids with **24a** was investigated. Pleasingly, it was found that in the case of the aryl boronic acids, good yields of the resultant styryl adducts **27–30** were observed. It was found that employment of the boronic acids in excess (approximately 5 equiv) in conjunction with base work-up gave the desired products in good yields with no starting material **24a**, or the product of bromine–hydrogen exchange **5a**. Unfortunately, under identical conditions no cross-coupled products were obtained following several attempts using *Z*-crotyl boronic acid and vinyl boronic acid.

**Figure 5:**
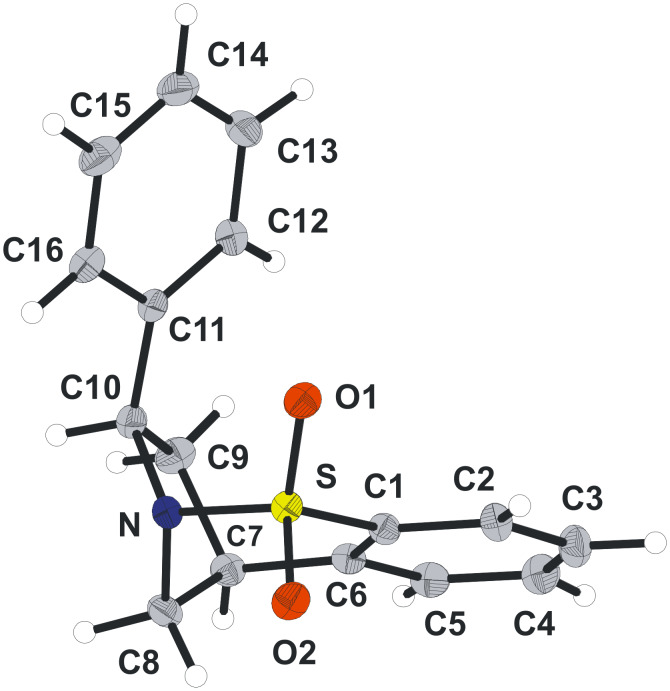
X-ray crystal structure of **31** (Diamond representation).

Hydrogenation of the trisubstituted alkene in compounds **27–30** was carried out in DMF due to the poor solubility of these compounds in more standard solvents. These reactions also proved rather slow. Nevertheless, after 72 h under a hydrogen atmosphere in the presence of Pd/C, moderate to good yields of compounds **31–34** were achieved. As predicted, the addition of hydrogen took place in a diastereoselective manner and the adducts were formed as single diastereoisomers. This sense of diastereoselectivity was confirmed by a series of NOE experiments and the single X-ray crystal structure obtained for compound **31** (see Figure 5 and crystallographic data). Poor conversion was observed when the one-pot reductive-palladium method, analogous to that described in Scheme 2, was attempted.

**Scheme 9:**
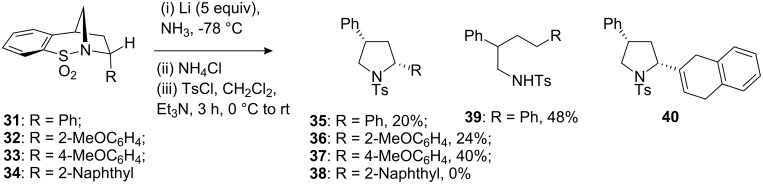
Attempted sulfonamide double reduction of compounds **31–34**.

The double reduction of compounds **31–34** was next considered. Cleavage of both the S–N and S–C bonds would result in the formation of a series of *cis*-diaryl-substituted pyrrolidine ring containing compounds. Consequently, treatment of each compound under the currently optimal conditions (lithium in liquid ammonia at −78 °C) gave the products of reduction which were converted into the corresponding tosyl derivatives to aid characterisation and purification (Scheme 9).

The successful double reduction of compounds **31–33** was observed in moderate/low yields. In the case of compound **31**, a significant amount of the product **39** of benzylic cleavage was also detected. This type of product was not observed for the more electron rich, methoxy-containing substituents, presumably since the Birch-type radical anionic intermediate, which enables the elimination of the amino group, is disfavoured in this instance. In the case of compound **34**, trace amount of the hoped for product **38** was detected. However, in this case the major product **40** stems from the partial reduction of the naphthyl ring in addition to cleavage of the sulfonyl tether.

## Conclusion

In summary, the work described demonstrates that we were able to utilise the functionalisation of bicyclic sulfonamide **5a** featuring a Suzuki coupling and a diastereoselective hydrogenation to construct *cis*-2,4-diarylpyrrolidines in a diastereoselective manner. The epimerisation reaction which led to the formation of carboxylic acid **26** demonstrates, in principle, how the *trans*-2,4-substituted series might also be accessible. The *cis*-diastereoselective dibromination of the bridgehead sultams **5a** and **5b** was also studied from a mechanistic perspective and related to this an unusual molecular somersault was uncovered, the occurrence of which was dependent on the electronic nature of the aryl motif and of the reaction solvent.

## Experimental

Representative experimental procedures are shown for compounds **7a**, **13a**, **18b**, **24a**, **27**, **31**, **35**, **39**. For full experimental details see accompanying Supporting Information.

**(±)-(1*S*,10*R*,11*R*)-Epoxy-8-thia-9-azatricyclo[7.2.1.0^2,7^]dodeca-2(7),3,5-triene-8,8-dioxide (7a):** To a solution of alkene **5a** (252 mg, 1.22 mmol, 1 equiv) in CH_2_Cl_2_ (50 mL) at 0 °C, 70% (w/w) *m*-CPBA (1.68 g, 6.82 mmol, 5.5 equiv) was added. The reaction mixture was stirred for 72 h during which time room temperature was reached. The reaction was quenched with a saturated Na_2_SO_3_ solution (15 mL) which was basified after 0.5 h with a saturated NaHCO_3_ solution (15 mL). H_2_O (10 mL) was added and the aqueous layer was then extracted with CH_2_Cl_2_ (2 × 20 mL). The combined organic extracts were dried over MgSO_4_. Filtration followed by solvent removal in vacuo afforded the crude product, which was purified by flash column chromatography (c-Hex–EtOAc; 5:1) affording **7a** (179 mg, 66%) as a colourless solid. Recrystallisation from EtOAc gave crystals suitable for X-ray crystallographic analysis. mp 164–165 °C (EtOAc); *R_f_* = 0.45 (c-Hex–EtOAc; 1:1); ^1^H NMR (300 MHz, CDCl_3_): δ 3.27 (d, *J* = 4.0 Hz, 1H, 1-CH), 3.35 (dd, *J* = 4.0, 12.5 Hz, 1H, 1H, 12a-CH_2_), 3.65 (s, 1H, 11-CH), 3.92 (d, *J* = 12.5 Hz, 1H, 12b-CH_2_), 5.03 (s, 1H, 10-CH), 7.19 (d, *J* = 6.5 Hz, 1H, ArH), 7.40–7.47 (m, 2H, ArH), 7.67–7.80 (m, 1H, ArH); ^13^C NMR (100 MHz, CDCl_3_): δ 39.5 (CH), 49.4 (CH_2_), 57.8 (CH), 65.4 (CH), 127.0 (CH), 127.7 (CH), 130.1 (CH), 132.5 (CH), 135.3 (C), 136.3 (C); υ_max_ (CH_2_Cl_2_/cm^−1^) (KCl) 3067, 3037, 1472, 1381, 1335, 1246, 1205, 1170, 1061, 1007, 935, 916, 855, 811, 792, 767, 750, 715, 609; *m*/*z* (ES) required 224.0379 (MH^+^, 100%); found 224.0381 (−1.1 ppm); Anal. Calcd for C_10_H_9_NO_3_S: C, 53.80; H, 4.06; N, 6.27. Found: C, 53.63; H, 4.04; N, 6.06.

**(±)-(1*S*,10*R*,11*R*)-10,11-Dibromo-8-thia-9-azatricyclo[7.2.1.0^2,7^]dodeca-2(7),3,5-triene-8,8-dioxide (13a)** [20]**:** To a solution of alkene **5a** (50 mg, 0.24 mmol, 1.2 equiv) in PhMe (10 mL) at −78 °C, Br_2_ (0.01 mL, 0.20 mmol, 1 equiv) was added. The reaction mixture was stirred for 15 h during which time, room temperature was reached. The reaction was quenched with a saturated solution of Na_2_SO_3_ (10 mL) and H_2_O (10 mL) was added. The resultant aqueous layer was then further extracted with CH_2_Cl_2_ (2 × 10 mL) and the combined organic extracts were dried over MgSO_4_. Filtration followed by solvent removal in vacuo afforded the crude product, which was purified by flash column chromatography (c-Hex–EtOAc; 9:1) affording **13a** (55 mg, 75%) as a colourless solid. mp 174–176 °C (EtOAc), lit. 185–188 °C [20]; *R_f_* = 0.6 (c-Hex–EtOAc; 1:1); ^1^H NMR (400 MHz, CDCl_3_): δ 3.66 (s, 1H, 1-CH), 4.10 (d, *J* = 13.0 Hz, 1H, 12a-CH_2_), 4.30 (d, *J* = 13.0 Hz, 1H, 12b-CH_2_), 4.66 (d, *J* = 6.0 Hz, 1H, 11-CH), 6.47 (d, *J* = 6.0 Hz, 1H, 10-CH), 7.35 (d, *J* = 7.0 Hz, 1H, ArH), 7.50–7.60 (m, 2H, ArH), 7.81 (d, *J* = 7.0 Hz, 1H, ArH); ^13^C NMR (100 MHz, CDCl_3_): δ 52.3 (CH), 52.8 (CH_2_), 57.8 (CH), 67.6 (CH), 126.6 (CH), 126.7 (CH), 130.5 (CH), 133.7 (CH), 134.9 (C), 135.9 (C); υ_max_ (CH_2_Cl_2_/cm^−1^) (NaCl) 3010, 2977, 1446, 1344, 1324, 1300, 1263, 1234, 1206, 1162, 1077; Anal. Calcd for C_10_H_9_Br_2_NO_2_S: C, 32.70; H, 2.45; N, 3.89; Br, 43.60. Found: C, 32.66; H, 2.31; N, 3.41; Br, 43.27.

**(±)-(1*R*,11*R*,12*S*)-11,12-Dibromo-4,5-dimethoxy-8-thia-9-azatricyclo[7.2.1.0^2,7^]dodeca-2(7),3,5-triene-8,8-dioxide (18b):** To a solution of alkene **5b** (75 mg, 0.28 mmol, 1 equiv) in CHCl_3_ (10 mL) at −78 °C, Br_2_ (0.15 mL, 2.80 mmol, 10 equiv) was added. The reaction mixture was stirred for 15 h during which time, room temperature was reached. The reaction was quenched with a saturated solution of Na_2_SO_3_ (20 mL) and H_2_O (20 mL) was added. The aqueous layer was then further extracted with CH_2_Cl_2_ (2 × 20 mL) and the combined organic extracts were dried over MgSO_4_. Filtration followed by solvent removal in vacuo afforded the crude product, which was purified by flash column chromatography (c-Hex–EtOAc; 5:1) affording **18b** (104 mg, 87%) as a colourless solid. Recrystallisation from EtOAc gave crystals suitable for X-ray crystallographic analysis. mp 225 °C (EtOAc); *R_f_* = 0.6 (c-Hex–EtOAc; 1:1); ^1^H NMR (500 MHz, CDCl_3_): δ 3.86 (s, 1H, 1-CH), 3.91 (s, 3H, CH_3_), 3.96 (s, 3H, CH_3_), 4.13 (dd, *J* = 5.0, 8.0 Hz, 1H, 11-CH), 4.20 (dd, *J* = 5.0, 15.0 Hz, 1H, 10a-CH_2_), 4.60 (dd, *J* = 8.0, 15.0 Hz, 1H, 10b-CH_2_), 6.32 (s, 1H, 12-CH), 6.70 (s, 1H, ArH), 7.19 (s, 1H, ArH); ^13^C NMR (125 MHz, CDCl_3_): δ 44.0 (CH), 56.3 (CH), 56.4 (CH_3_), 58.5 (CH_3_), 58.9 (CH), 64.5 (CH_2_), 107.8 (CH), 108.5 (CH), 126.3 (C), 130.2 (C), 150.4 (C), 153.2 (C); υ_max_ (CH_2_Cl_2_/cm^−1^) (NaCl) 3096, 2975, 1593, 1444, 1326, 1260, 1203, 1164, 1044; Anal. Calcd for C_12_H_13_Br_2_NO_4_S: C, 33.75; H, 3.07; N, 3.28. Found: C, 33.69; H, 2.99; N, 3.17.

**10-Bromo-8-thia-9-azatricyclo[7.2.1.0^2,7^]dodeca-2,4,6,10-tetraene-8,8-dioxide (24a)** [20]**:** Compound **13a** (1.04 g, 2.83 mmol, 1 equiv) in THF (15 mL) was treated with a 1 M solution of TBAF in THF (20 mL, 20.00 mmol, 7 equiv) for 15 h. The solvent was removed in vacuo and CH_2_Cl_2_ was added (30 mL). The organic layer was washed with a saturated solution of NaHCO_3_ (30 mL), the aqueous phase further extracted with CH_2_Cl_2_ (30 mL) and the combined organic layers were dried over MgSO_4_. Filtration followed by solvent removal in vacuo afforded the crude product, which was purified by flash column chromatography (c-Hex–EtOAc; 1:1) affording **24a** (700 mg, 86%) as a colourless solid. mp 188–192 °C (CH_2_Cl_2_); *R_f_* = 0.3 (c-Hex–EtOAc; 1:1); ^1^H NMR (400 MHz, CDCl_3_): δ 3.29 (t, *J* = 4.0 Hz, 1H, CH), 4.37 (dd, *J* = 4.0, 12.0 Hz, 1H, 12a-CH_2_), 4.59 (d, *J* = 12.0 Hz, 1H, 1H, 12b-CH_2_), 6.68 (d, *J* = 4.0 Hz, 1H, 11-CH), 7.12 (d, *J* = 7.5 Hz, 1H, ArH), 7.42 (t, *J* = 7.5 Hz, 1H, ArH), 7.50 (t, *J* = 7.5 Hz, 1H, ArH), 7.77 (d, *J* = 7.5 Hz, 1H, ArH); ^13^C NMR (100 MHz, CDCl_3_): δ 43.9 (CH), 65.5 (CH_2_), 125.0 (C), 125.4 (CH), 127.4 (CH), 130.2 (CH), 132.1 (CH), 134.5 (C), 135.9 (CH), 139.3 (C); υ_max_ (CH_2_Cl_2_/cm^−1^) (NaCl) 3102, 2924, 1591, 1452, 1338, 1168, 1054, 925, 866, 749; *m*/*z* (ES) required 285.9549 (MH^+^(Br^79^), 100%); found 285.9537 (+4.1 ppm); Anal. Calcd for C_10_H_8_BrNO_2_S: C, 41.96; H, 2.80; N, 4.90; Found: C, 41.97; H, 2.77; N, 4.71.

**10-Phenyl-8-thia-9-azatricyclo[7.2.1.0^2,7^]dodeca-2,4,6,10-tetraene-8,8-dioxide (27):** Under N_2_, a mixture of the compound **24a** (500 mg, 1.75 mmol, 1 equiv), phenylboronic acid (1.073 g, 8.80 mmol, 5 equiv), Pd(OAc)_2_ (20 mg, 0.09 mmol, 5 mol %), PPh_3_ (47 mg, 0.18 mmol, 10 mol %) and Cs_2_CO_3_ (1.714 g, 5.26 mmol, 3 equiv) in a mixture of THF:H_2_O (10:1) (25 mL) was heated to reflux for 15 h. On cooling Et_2_O (20 mL) and H_2_O (20 mL) were added and the resultant aqueous layer was further extracted with Et_2_O (2 × 20 mL) and the combined organic extracts were washed with a 2 M NaOH solution (20 mL) and dried over MgSO_4_. Filtration followed by solvent removal under reduced pressure gave the crude product, which was purified by flash column chromatography (c-Hex–EtOAc; 5:1) affording **27** (314 mg, 63%) as a colourless solid. mp 120–122 °C (EtOAc); *R_f_* = 0.55 (c-Hex–EtOAc; 1:1); ^1^H NMR (400 MHz, CDCl_3_): δ 3.44 (t, *J* = 4.0 Hz, 1H, CH), 4.27 (dd, *J* = 4.0, 12.0 Hz, 1H, CH_2_), 4.70 (d, *J* = 12.0 Hz, 1H, CH_2_), 6.82 (d, *J* = 4.0 Hz, 1H, CH), 7.17 (d, *J* = 7.5 Hz, 1H, ArH), 7.31–7.39 (m, 3H, ArH), 7.40–7.47 (m, 2H, ArH), 7.69–7.71 (m, 3H, ArH); ^13^C NMR (100 MHz, CDCl_3_): δ 43.3 (CH), 64.3 (CH_2_), 110.0 (C), 125.3 (CH), 126.6 (CH), 127.1 (CH), 127.3 (CH), 128.3 (CH), 129.2 (CH), 129.7 (CH), 131.7 (CH), 134.7 (C), 140.8 (C), 147.0 (C); υ_max_ (CH_2_Cl_2_/cm^−1^) (KCl) 3063, 1965, 1592, 1452, 1327, 1162, 1029, 948; *m*/*z* (ES) required 284.0733 (MH^+^, 100%); found 284.0745 (−4.3 ppm); Anal. Calcd for C_16_H_13_NO_2_S: C, 67.84; H, 4.59; N, 4.95. Found: C, 67.69; H, 4.65; N, 4.84.

**(±)-(1*S*,10*R*)-10-Phenyl-8-thia-9-azatricyclo[7.2.1.0^2,7^]dodeca-2(7),3,5-triene-8,8-dioxide (31):** 10% (w/w) Pd/C (98 mg, 0.09 mmol, 15 mol %) was added to a solution of the alkene **27** (173 mg, 0.61 mmol, 1 equiv) in DMF (20 mL). The mixture was degassed before stirring under a hydrogen atmosphere (1 atm) at room temperature for 72 h. Filtration through Celite^®^ and solvent removal in vacuo gave **32** (77 mg, 44%) as a colourless crystalline solid. mp 131–134 °C (EtOAc); *R_f_* = 0.5 (c-Hex–EtOAc; 1:1); ^1^H NMR (400 MHz, CDCl_3_): δ 2.36 (ddd, *J* = 2.0, 7.5, 13.0 Hz, 1H, CH_2_), 2.73 (ddd, *J* = 7.5, 10.0, 13.0 Hz, 1H, CH_2_), 3.43 (dd, *J* = 3.5, 7.5 Hz, 1H, CH), 3.56 (dd, *J* = 3.5, 12.5 Hz, 1H, CH_2_), 4.53 (dd, *J* = 2.0, 12.5 Hz, 1H, CH_2_), 5.05 (dd, *J* = 7.5, 10.0 Hz, 1H, CH), 7.13–7.19 (m, 2H, ArH), 7.22–7.29 (m, 4H, ArH), 7.36 (dt, *J* = 1.0, 7.0 Hz, 1H, ArH), 7.48 (dt, *J* = 1.0, 7.5 Hz, 1H, ArH), 7.66 (d, *J* = 7.5 Hz, 1H, 1H, ArH); ^13^C NMR (100 MHz, CDCl_3_): δ 37.7 (CH_2_), 40.1 (CH), 59.1 (CH_2_), 66.4 (CH), 125.8 (CH), 127.0 (CH), 127.9 (CH), 128.2 (CH), 128.3 (CH), 129.8 (CH), 132.6 (CH), 135.1 (C), 137.2 (C), 142.1 (C); υ_max_ (CH_2_Cl_2_/cm^−1^) (KCl) 3042, 2956, 2342, 1954, 1876, 1596, 1452, 1324, 1163, 1087, 975; *m*/*z* (ES) required 284.0894 (MH^+^, 100%); found 284.0902 (−2.7 ppm).

**(±)-(2*R*,4*S*)-2,4-Diphenyl-1-(toluene-4-sulfonyl)pyrrolidine (35) and** ***N*-(2,4-diphenylbutyl)-4-methylbenzenesulfonamide (39):** Under N_2_ at −78 °C liquid NH_3_ (ca. 100 mL) was treated with lithium wire (10 mg, 1.43 mmol, 5 equiv). This mixture was stirred for 1 h before a solution of **31** (80 mg, 0.28 mmol, 1 equiv) in THF (5 mL) was introduced dropwise. Stirring was continued at −78 °C for 20 min before solid NH_4_Cl (ca. 5 g) was added. The NH_3_ was allowed to evaporate and Et_2_O (25 mL) and H_2_O (25 mL) were added to the residue. The resultant aqueous layer was further extracted with Et_2_O (2 × 25 mL) and the combined organic extracts were dried over MgSO_4_. Filtration, followed by solvent removal under reduced pressure, afforded a colourless oil. At 0 °C the crude material was treated with Et_3_N (0.06 mL, 0.43 mmol, 1.5 equiv) and TsCl (53 mg, 0.28 mmol, 1 equiv) in CH_2_Cl_2_ (10 mL). The mixture was stirred for 3 h during which time, room temperature was reached. CH_2_Cl_2_ (20 mL) and H_2_O (20 mL) were added and the resultant aqueous layer was further extracted with CH_2_Cl_2_ (2 × 20 mL). The combined organic extracts were dried over MgSO_4_, which was removed by filtration. The solvent was removed under reduced pressure and purification of the residue by flash column chromatography (c-Hex–EtOAc; 4:1) gave initially **35** (21 mg, 20%) as a colourless oil. *R_f_* = 0.45 (c-Hex–EtOAc; 3:1); ^1^H NMR (400 MHz, CDCl_3_): δ 2.05 (ddd, app. dt, *J* = 10.0, 12.5 Hz, 1H, CH_2_), 2.43 (s, 3H, CH_3_), 2.63–2.72 (m, 1H, CH_2_), 2.90–3.02 (m, 1H, CH), 3.51 (t, *J* = 11.0 Hz, 1H, CH_2_), 4.16 (ddd, *J* = 1.0, 7.0, 11.0 Hz, 1H, CH_2_), 4.82 (dd, *J* = 7.0, 10.0 Hz, 1H, CH), 7.12 (d, *J* = 7.0 Hz, 2H, ArH), 7.20–7.36 (m, 10H, ArH), 7.63 (d, *J* = 8.0 Hz, 2H, ArH); ^13^C NMR (100 MHz, CDCl_3_): δ 21.5 (CH_3_), 43.7 (CH), 44.4 (CH_2_), 55.9 (CH_2_), 64.5 (CH), 126.4 (CH), 127.0 (CH), 127.1 (CH), 127.3 (CH), 127.4 (CH), 128.4 (CH), 128.7 (CH), 129.6 (CH), 135.7 (C), 139.0 (C), 142.5 (C), 143.3 (C); υ_max_ (CH_2_Cl_2_/cm^−1^) 3059, 2926, 1599, 1494, 1432, 1342, 1289, 1254, 1158, 1093, 1027; *m*/*z* (ES) required 378.1535 (MH^+^, 100%); found 378.1528 (+1.1 ppm). Further elution gave **39** (51 mg, 48%) as a colourless oil. *R_f_* = 0.35 (c-Hex–EtOAc; 3:1); ^1^H NMR (300 MHz, CDCl_3_): δ 1.77–1.88 (m, 1H, CH_2_), 1.90–2.00 (m, 1H, CH_2_), 2.34–2.49 (m, 5H, CH_3_, CH_2_), 2.63–2.72 (m, 1H, CH), 3.00 (app. ddt, *J* = 4.5, 9.0 Hz, 1H, CH_2_), 3.27 (app. ddt, *J* = 5.5, 8.0 Hz, 1H, CH_2_), 4.21 (dd, *J* = 4.0, 4.5 Hz, 1H, NH), 7.01–7.06 (m, 4H, ArH), 7.13–7.35 (m, 8H, ArH), 7.62 (d, *J* = 8.0 Hz, 2H, ArH); ^13^C NMR (100 MHz, CDCl_3_): δ 21.5 (CH_3_), 33.1 (CH_2_), 35.0 (CH_2_), 45.0 (CH), 48.5 (CH_2_), 125.9 (CH), 127.0 (CH), 127.3 (CH), 127.8 (CH), 128.3 (CH), 128.35 (CH), 129.0 (CH), 129.6 (CH), 137.0 (C), 141.0 (C), 141.5 (C), 143.3 (C); υ_max_ (CH_2_Cl_2_/cm^−1^) 3360, 2043, 2932, 1599, 1493, 1431, 1327, 1222, 1159, 1087, 810; *m*/*z* (ES) required 380.1666 (MH^+^, 100%); found 380.1684 (−4.8 ppm).

### Crystallographic data

Crystal structural data for compound **7a**: C_10_H_9_NO_3_S; *M* = 223.24; orthorhombic, *Pbca*; *a* = 8.9434(12) Å, *b* = 12.1447(16) Å, *c* = 17.0400(2) Å; *U* = 1850.8(4) Å^3^; *T* = 100(2) K; *Z* = 8; 15241 reflections measured, 2019 unique (*R*_int_ = 0.0514). The final *wR*^2^ was 0.1169 (all data). CCDC reference number 742466. Crystal structure data for compound **13a**: C_10_H_9_NO_2_SBr_2_; *M* = 367.06; monoclinic, *P*2_1_/*c*; *a* = 11.1268(11) Å, *b* = 8.6567(8) Å, *c* = 12.5715(12) Å; *U* = 1129.8(19) Å^3^; *T* = 100(2) K; *Z* = 4; 11016 reflections measured, 2808 unique (*R*_int_ = 0.0407). The final *wR*^2^ was 0.0671 (all data). CCDC reference number 742467. Crystal structural data for compound **18b**: C_12_H_13_NO_4_SBr_2_; *M* = 427.11; monoclinic, *P*2_1_/*n*; *a* = 11.462(2) Å, *b* = 10.4508(18) Å, *c* = 12.0450(2) Å; *U* = 1405.2(4) Å^3^; *T* = 100(2) K; *Z* = 4; 11889 reflections measured, 2867 unique (*R*_int_ = 0.0378). The final *wR*^2^ was 0.0946 (all data). CCDC reference number 742468. Crystal structural data for compound **19b**: C_13_H_16_NO_5_SBr; *M* = 378.24; monoclinic, *P*2_1_/*c*; *a* = 15.9581(19) Å, *b* = 10.6318(13) Å, *c* = 17.8100(2) Å; *U* = 2889.5(6) Å^3^; *T* = 100(2) K; *Z* = 8; 65857 reflections measured, 8789 unique (*R*_int_ = 0.0329). The final *wR*^2^ was 0.0712 (all data). CCDC reference number 742469. Crystal structural data for compound **26**: C_11_H_11_NO_4_S; *M* = 253.27; monoclinic, *P*2_1_/*n*; *a* = 14.7519(16) Å, *b* = 9.6485(10) Å, *c* = 15.2476(16) Å; *U* = 2163.8(4) Å^3^; *T* = 100(2) K; *Z* = 8; 37265 reflections measured, 4487 unique (*R*_int_ = 0.0384). The final *wR*^2^ was 0.1158 (all data). CCDC reference number 742471. Crystal structural data for compound **31**: C_16_H_15_NO_2_S; *M* = 285.35; orthorhombic, *P*2_1_2_1_2_1_; *a* = 9.3949(7) Å, *b* = 10.7576(8) Å, *c* = 13.5221(10) Å; *U* = 1366.63(18) Å^3^; *T* = 180(2) K; *Z* = 4; 16599 reflections measured, 4468 unique (*R*_int_ = 0.0157). The final *wR*^2^ was 0.0852 (all data). CCDC reference number 742470.

## Supporting Information

Supporting information features experimental procedures and spectroscopic analyses for compounds **6a**, **6b**, **7a**, **7b**, **13a**, **13b**, **16a**, **16b**, **17a**, **18b**, **19b**, **24a**, **24b**, **25–37**, and **39**.

File 1 Diastereoselective functionalisation of benzo-annulated bicyclic sultams: application for the synthesis of *cis*-2,4-diarylpyrrolidines.
