# Supplementary material for: Diastereoselective functionalisation of benzo-annulated bicyclic sultams: Application for the synthesis of cis-2,4-diarylpyrrolidines
Source: Beilstein J Org Chem. 2009 Nov 25;5:69. doi: 10.3762/bjoc.5.69 (PMC2839534; doi:10.3762/bjoc.5.69)
Supplement: File 1 — Diastereoselective functionalisation of benzo-annulated bicyclic sultams: application for the synthesis of cis-2,4-diarylpyrrolidines. [file Beilstein_J_Org_Chem-05-69-s001.pdf]

Supporting Information

Diastereoselective functionalisation of benzo-annulated bicyclic sultams: Application for the synthesis of *cis*-2,4-diarylpyrrolidines

Susan Kelleher, Pierre-Yves Quesne and Paul Evans*
School of Chemistry and Chemical Biology, University College Dublin, Dublin 4, Ireland. Email: paul.evans@ucd.ie

Experimental procedures

General directions

Reagents were obtained from commercial suppliers unless otherwise stated. Dry THF and diethyl ether were distilled from sodium benzophenone ketyl radical and dry CH_2Cl_2 distilled from calcium hydride, all under nitrogen. Low reaction temperatures were obtained with an acetone/solid CO_2 bath. Thin-layer chromatography was performed on silica coated aluminium sheets (60 F_{254}) supplied by Merck. Flash column chromatography was performed using flash silica 60 Å (230–400 mesh) 9385 supplied by Merck. ^{1}H and ^{13}C NMR spectra were recorded using Varian Inova 300 MHz, 400 MHz and 500 MHz instruments. Deuterochloroform was used as the solvent and chemical shifts are given in ppm relative to the standard reference TMS or residual chloroform. Infrared spectra were recorded on a Mattson Instruments Galaxy series FT-IR 3000 spectrometer. Melting points were recorded on a Gallenkamp electrothermal melting point apparatus and are uncorrected. High resolution mass spectra were carried out on a VG analytical 70-E mass spectrometer. The names of compounds provided were generated using the Autonom programme on the Beilstein chemical database. Compounds **5a** and **5b** were prepared according to the reported procedures.[1]

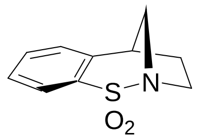

8-Thia-9-azatricyclo[7.2.1.$0^{2,7}$]dodeca-2(7),3,5-triene-8,8-dioxide (6a): Under N_2, a mixture of the dihydropyrrole **4a** (160 mg, 0.56 mmol, 1 equiv), $Pd(OAc)_2$ (14 mg, 0.06 mmol, 11 mol%), PPh_3 (25 mg, 0.10 mmol, 17 mol%) and K_2CO_3 (154 mg, 1.11 mmol, 2 equiv) in DMF (20 mL) was heated to 110 °C for 15 h. On cooling H_2 was bubbled through the reaction for 1 h. The reaction vessel was then stirred under a H_2 atmosphere for 15 h. Et_2O (25 mL) and H_2O (25 mL) were added and the resultant aqueous layer was further washed with Et_2O (4 × 25 mL) and the combined organic layers were dried over $MgSO_4$. Filtration followed by solvent removal under reduced pressure gave the crude product which was purified by flash column chromatography (c-Hex–EtOAc, 5:1) affording the product **6a** (71 mg, 61%) as a colourless crystalline solid with data as previously reported. mp 119–121 °C.[1]

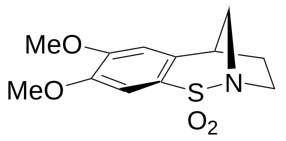

4,5-Dimethoxy-8-thia-9-azatricyclo[7.2.1.$0^{2,7}$]dodeca-2(7),3,5-triene-8,8-dioxide (6b): As above, a mixture of the dihydropyrrole **4b** (1.50 g, 4.31 mmol, 1 equiv), $Pd(OAc)_2$ (97 mg, 0.43 mmol, 10 mol%), PPh_3 (225 mg, 0.86 mmol, 20 mol%) and K_2CO_3 (1.19 g, 8.61 mmol, 2 equiv) in DMF (60 mL) was heated to 110 °C for 36 h under N_2. On cooling H_2 bubbled through the reaction for 1 h. The reaction was stirred under H_2 for 15 h. Et_2O (50 mL) and H_2O (50 mL) were added. The resultant aqueous layer was further washed with Et_2O (4 × 50 mL) and the combined organic layers were dried over $MgSO_4$. Filtration followed by solvent removal under reduced pressure gave the crude product which was purified by flash column chromatography (c-Hex–EtOAc, 3:1) affording the product **3b** (600 mg, 52%) as a colourless solid with data as previously reported. mp 198–200 °C.[1]

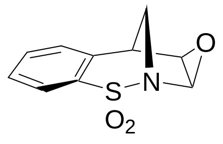

(±)-(1*S*,10*R*,11*R*)-Epoxy-8-thia-9-azatricyclo[7.2.1.$0^{2,7}$]dodeca-2(7),3,5-triene-8,8-dioxide (7a): To a solution of alkene **5a** (252 mg, 1.22 mmol, 1 equiv) in CH_2Cl_2 (50 mL) at 0 °C was added 70% (w/w) *m*-CPBA (1.68 g, 6.82 mmol, 5.5 equiv) and the reaction mixture was allowed to stir for 72 h during which time room temperature was reached. The reaction was quenched with a saturated

Na_2SO_3 solution (15 mL) which was basified after 0.5 h with a saturated $NaHCO_3$ solution (15 mL). H_2O (10 mL) was added and the aqueous layer was then extracted with CH_2Cl_2 (2 × 20 mL). The combined organic extracts were dried over $MgSO_4$. Filtration followed by solvent removal in vacuo afforded the crude product, which was purified by flash column chromatography (c-Hex–EtOAc, 5:1) affording **7a** (179 mg, 66%) as a colourless solid. Recrystallisation from EtOAc gave crystals suitable for X-ray crystallographic analysis. mp 164–165 °C (EtOAc); R_f = 0.45 (c-Hex–EtOAc, 1:1); ^{1}H NMR (300 MHz, $CDCl_3$): δ 3.27 (d, *J* = 4.0 Hz, 1H, 1-CH), 3.35 (dd, *J* = 4.0, 12.5 Hz, 1H, 1H, 12a-CH_2), 3.65 (s, 1H, 11-CH), 3.92 (d, *J* = 12.5 Hz, 1H, 12b-CH_2), 5.03 (s, 1H, 10-CH), 7.19 (d, *J* = 6.5 Hz, 1H, ArH), 7.40–7.47 (m, 2H, ArH), 7.67–7.80 (m, 1H, ArH); ^{13}C NMR (100 MHz, $CDCl_3$): δ 39.5 (CH), 49.4 (CH_2), 57.8 (CH), 65.4 (CH), 127.0 (CH), 127.7 (CH), 130.1 (CH), 132.5 (CH), 135.3 (C), 136.3 (C); υ_{max} (CH_2Cl_2/cm^{-1}) (KCl) 3067, 3037, 1472, 1381, 1335, 1246, 1205, 1170, 1061, 1007, 935, 916, 855, 811, 792, 767, 750, 715, 609; *m*/*z* (ESI) required 224.0379 (MH^+, 100%); found 224.0381 (−1.1 ppm); Anal. Calcd for $C_{10}H_9NO_3S$: C, 53.80; H, 4.06; N, 6.27. Found: C, 53.63; H, 4.04; N, 6.06.

Alternative method: To a solution of alkene **5a** (146 mg, 0.71 mmol, 1 equiv) in CH_3CN (25 mL) was added an aqueous Na_2EDTA solution (1.2 mM, 0.83 mL, 0.001 mmol, 0.15 mol%). The resulting solution was cooled to 0 °C before addition of F_3CCOCH_3 (1.08 mL, 12.07 mmol, 17 equiv) via a pre-cooled syringe. To this solution was added a mixture of sodium bicarbonate (462 mg, 5.50 mmol, 8 equiv) and oxone (2.15 g, 7.00 mmol, 10 equiv) in portions over 1 h. The reaction was stirred and allowed to warm to room temperature overnight. CH_2Cl_2 (15 mL) and H_2O (15 mL) were added and the resultant aqueous layer was further extracted with CH_2Cl_2 (2 × 15 mL). The combined organic extracts were dried over $MgSO_4$. Filtration followed by solvent removal under reduced pressure gave **7a** (152 mg, 96%) as a colourless solid with data as above.

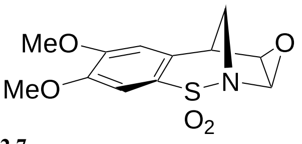

(±)-(1*S*,10*R*,11*R*)-4,5-Dimethoxy-10,11-epoxy-8-thia-9-azatricyclo[7.2.1.0^{2,7}]dodeca-2(7),3,5-triene-8,8-dioxide (7b): To a solution of alkene **5b** (500 mg, 1.87 mmol, 1 equiv) in CH_2Cl_2 (100 mL) at 0 °C was added 70% (w/w) *m*-CPBA (2.74 g, 11.12 mmol, 6 equiv). The reaction was stirred for 15 h during which time room temperature was reached. A saturated solution of Na_2SO_3 (50 mL) was added. After 0.5 h a saturated solution of $NaHCO_3$ (50 mL) was added. The aqueous layer was then extracted with CH_2Cl_2 (2 × 75 mL) and the combined organic extracts were dried over $MgSO_4$. Filtration followed by solvent removal in vacuo afforded the crude product, which was purified by flash column chromatography (c-Hex–EtOAc, 5:1) affording **7b** (430 mg, 81%) as a colourless solid. mp 227–230 °C; R_f = 0.35 (c-Hex–EtOAc, 1:1); ^{1}H NMR (400 MHz, $CDCl_3$): δ 3.30 (d, *J* = 3.5 Hz, 1H, 1-CH), 3.40 (dd, *J* = 3.5, 12.5 Hz, 1H, 12a-CH_2), 3.71 (s, 1H, 11-CH), 3.91 (s, 3H, CH_3), 3.93 (s, 3H, CH_3), 3.90–3.99 (d, *J* = 12.5 Hz, 1H, 12b-CH_2), 5.08 (s, 1H, 10-CH), 6.68 (s, 1H, ArH), 7.21 (s, 1H, ArH); ^{13}C NMR (100 MHz, $CDCl_3$): δ 39.3 (CH), 49.6 (CH_2), 56.2 (CH_3), 56.3 (CH_3), 57.9 (CH), 65.5 (CH), 108.7 (CH), 109.6 (CH), 127.3 (C), 128.8 (C), 150.0 (C), 151.9 (C); υ_{max} (CH_2Cl_2/cm^{-1}) (NaCl) 3092, 3019, 2967, 2929, 2846, 1660, 1599, 1507, 1463, 1438, 1401, 1326, 1264, 1225, 1147, 1056, 964, 905, 853, 811, 726, 771; *m*/*z* (ESI) required 282.0447 (M−H^+, 100%); found 282.0436 (+3.8 ppm); Anal. Calcd for $C_{12}H_{13}NO_5S$: C, 50.88; H, 4.59; N, 4.94. Found: C, 51.14; H, 4.79; N, 4.54.

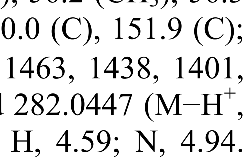

(±)-(1*S*,10*R*,11*R*)-10,11-Dibromo-8-thia-9-azatricyclo[7.2.1.0^{2,7}]dodeca-2(7),3,5-triene-8,8-dioxide (13a): To a solution of alkene **5a** (50 mg, 0.24 mmol, 1.2 equiv) in PhMe (10 mL) at −78

°C was added Br_2 (0.01 mL, 0.20 mmol, 1 equiv). The reaction mixture was left stirring for 15 h during which time room temperature was reached. The reaction was quenched with a saturated solution of Na_2SO_3 (10 mL) and H_2O (10 mL) was added. The resultant aqueous layer was then further extracted with CH_2Cl_2 (2 × 10 mL) and the combined organic extracts were dried over $MgSO_4$. Filtration followed by solvent removal in vacuo afforded the crude product, which was purified by flash column chromatography (c-Hex–EtOAc, 9:1) affording **13a** (55 mg, 75%) as a colourless solid. mp 174–176 °C (EtOAc), lit. 185–188 °C;[2] R_f = 0.6 (c-Hex–EtOAc, 1:1). ^{1}H NMR (400 MHz, $CDCl_3$): δ 3.66 (s, 1H, 1-CH), 4.10 (d, J = 13.0 Hz, 1H, 12a-CH_2), 4.30 (d, J = 13.0 Hz, 1H, 12b-CH_2), 4.66 (d, J = 6.0 Hz, 1H, 11-CH), 6.47 (d, J = 6.0 Hz, 1H, 10-CH), 7.35 (d, J = 7.0 Hz, 1H, ArH), 7.50–7.60 (m, 2H, ArH), 7.81 (d, J = 7.0 Hz, 1H, ArH); ^{13}C NMR (100 MHz, $CDCl_3$): δ 52.3 (CH), 52.8 (CH_2), 57.8 (CH), 67.6 (CH), 126.6 (CH), 126.7 (CH), 130.5 (CH), 133.7 (CH), 134.9 (C), 135.9 (C); υ_{max} (CH_2Cl_2/cm^{-1}) (NaCl) 3010, 2977, 1446, 1344, 1324, 1300, 1263, 1234, 1206, 1162, 1077; Anal. Calcd for $C_{10}H_9Br_2NO_2S$: C, 32.70; H, 2.45; N, 3.89; Br, 43.60. Found: C, 32.66; H, 2.31; N, 3.41; Br, 43.27.

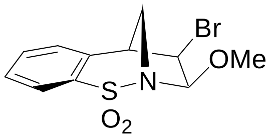

(±)-(1*S*,10*R*,11*R*)-11-Bromo-10-methoxy-8-thia-9-azatricyclo[7.2.1.0^{2,7}]dodeca-2(7),3,5-triene-8,8-dioxide (16a): To a solution of alkene **5a** (200 mg, 0.97 mmol, 1 equiv) in MeOH (20 mL) at −78 °C was added a Br_2 solution in MeOH (3 M, 0.33 mL, 0.99 mmol, 1 equiv) over 0.5 h. The reaction was left stirring for 15 h during which time room temperature was reached. The reaction was quenched with a saturated solution of Na_2SO_3 (15 mL) and H_2O (20 mL) was added. The aqueous layer was then further extracted with CH_2Cl_2 (2 × 20 mL) and the combined organic extracts were dried over $MgSO_4$. Filtration followed by solvent removal in vacuo afforded the crude product, which was purified by flash column chromatography (c-Hex–EtOAc, 5:1) affording **16a** (246 mg, 80%) as a colourless solid. mp 116–118 °C (CH_2Cl_2); R_f = 0.7 (c-Hex–EtOAc, 1:1); ^{1}H NMR (400 MHz, $CDCl_3$): δ 3.58 (s(br), 4H, CH, CH_3), 3.92 (dd, J = 2.5, 13.0 Hz, 1H, 12a-CH_2), 4.20 (d, J = 13.0 Hz, 1H, 12b-CH_2), 4.51 (d, J = 5.0 Hz, 1H, 11-CH), 5.15 (d, J = 5.0 Hz, 1H, 10-CH), 7.33 (d, J = 7.0 Hz, 1H, ArH), 7.46–7.56 (m, 2H, ArH), 7.76 (d, J = 7.0 Hz, 1H, ArH); ^{13}C NMR (100 MHz, $CDCl_3$): δ 51.7 (CH), 52.0 (CH_2), 54.2 (CH), 57.5 (CH_3), 91.2 (CH), 126.1 (CH), 128.6 (CH), 130.1 (CH), 133.3 (CH), 135.4 (C), 136.6 (C); υ_{max} (CH_2Cl_2/cm^{-1}) (NaCl) 3068, 3006, 2975, 2928, 2833, 1594, 1570, 1471, 1442, 1381, 1320, 1302, 1279, 1250, 1200, 1166, 1126, 1075, 1046; *m*/*z* (ESI) required 317.9815 (MH^+(Br^{79}), 100%); found 317.9800 (+4.9 ppm); Anal. Calcd for $C_{11}H_{12}BrNO_3S$: C, 41.51; H, 3.77; N, 4.40; Br, 25.16. Found: C, 41.25; H, 3.81; N, 4.24; Br, 24.71.

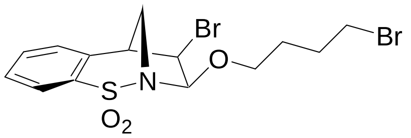

(±)-(1*S*,10*R*,11*R*)-11-Bromo-10-(4-bromobutoxy)-8-thia-9-azatricyclo[7.2.1.0^{2,7}]dodeca-2(7),3,5-triene-8,8-dioxide (17a): To a solution of alkene **5a** (50 mg, 0.24 mmol, 1 equiv) in THF (5 mL) at −78 °C was added a Br_2 solution in THF (20 M, 1.2 mL, 0.24 mmol, 1 equiv). The reaction was stirred for 15 h during which time room temperature was reached. The reaction was quenched with a saturated solution of Na_2SO_3 (15 mL) and H_2O (10 mL) was added. The aqueous layer was then further extracted with CH_2Cl_2 (2 × 20 mL) and the combined organic extracts were dried over $MgSO_4$. Filtration followed by solvent removal in vacuo afforded the crude product, which was purified by flash column chromatography (c-Hex–EtOAc, 5:1) affording **17a** (30 mg, 28%) as a colourless oil. R_f = 0.4 (c-Hex–EtOAc, 3:1); ^{1}H NMR (400 MHz, $CDCl_3$): δ 1.79–1.86 (m, 2H, 2'-CH_2), 1.90–2.05 (m, 2H, 3'-CH_2), 3.40–3.50 (m, 2H, 4'-CH_2), 3.57 (s, 1H, 1-CH), 3.62–3.69 (m, 1H, 1a'-CH_2), 3.95 (d, J = 13.0 Hz, 1H, 12a-CH_2), 3.96–4.01 (m, 1H, 1b'-CH_2), 4.32 (d, J = 13.0 Hz, 1H, 12b-CH_2), 4.51 (d, J = 5.5 Hz, 1H, 11-CH), 5.25 (d, J = 5.5 Hz, 1H, 10-CH), 7.33

(d, J = 7.0 Hz, 1H, ArH), 7.48–7.55 (m, 2H, ArH), 7.78 (d, J = 7.0 Hz, 1H, ArH); ^{13}C NMR (100 MHz, $CDCl_3$): δ 27.6 (CH_2), 29.8 (CH_2), 33.7 (CH_2), 51.8 (CH), 52.1 (CH_2), 54.5 (CH), 69.0 (CH_2), 89.7 (CH), 126.1 (CH), 128.6 (CH), 130.1 (CH), 133.3 (CH), 135.4 (C), 136.8 (C); υ_{max} (CH_2Cl_2/cm^{-1}) (NaCl) 1641, 1474, 1445, 1338, 1298, 1250, 1215, 1172, 1129, 1077, 1046; *m*/*z* (ESI) required 437.9395 (MH^+(Br^{79},Br^{79}), 30%); found 437.9374 (+4.8 ppm).

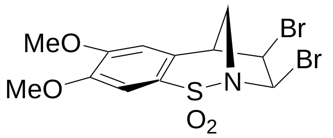

(±)-(1*R*,10*R*,11*R*)-10,11-Dibromo-4,5-dimethoxy-8-thia-9-azatricyclo[7.2.1.0^{2,7}]dodeca-2(7),3,5-triene-8,8-dioxide (13b): To a solution of alkene **5b** (80 mg, 0.30 mmol, 1 equiv) in PhMe (20 mL) at −78 °C was added a dilute solution of Br_2 in PhMe (0.3 M, 1.0 mL, 0.30 mmol, 1 equiv). The reaction was stirred for 15 h during which time room temperature was reached. A saturated solution of Na_2SO_3 (20 mL) followed by H_2O (20 mL) was added. The resultant aqueous layer was further extracted with CH_2Cl_2 (2 × 20 mL) and the combined organic extracts dried over $MgSO_4$. Filtration followed by solvent removal in vacuo afforded the crude product, which was purified by flash column chromatography (c-Hex–EtOAc, 5:1) affording **13b** (100 mg, 78%) as a colourless solid. mp 176–180 °C; R_f = 0.5 (c-Hex–EtOAc, 1:1); ^{1}H NMR (500 MHz, $CDCl_3$): δ 3.54 (s, 1H, 1-CH), 3.91 (s, 3H, CH_3), 3.95 (s, 3H, CH_3), 4.07 (dd, J = 3.0, 13.0 Hz, 1H, 11-CH), 4.26 (d, J = 12.0 Hz, 1H, 12a-CH_2), 4.65 (dd, J = 2.0, 6.0 Hz, 1H, 12b-CH_2), 6.72 (d, J = 6.0 Hz, 1H, 10-CH), 6.72 (s, 1H, ArH), 7.19 (s, 1H, ArH); ^{13}C NMR (100 MHz, $CDCl_3$): δ 56.3 (CH), 56.4 (CH_2), 58.5 (2 × CH_3), 58.9 (CH), 64.5 (CH), 107.8 (CH), 108.5 (CH), 126.3 (C), 130.2 (C), 150.4 (C), 153.2 (C); υ_{max} (CH_2Cl_2/cm^{-1}) (NaCl) 3020, 2975, 1593, 1444, 1326, 1260, 1203, 1164, 1044, 978; *m*/*z* (ESI) required 425.9008 (MH^+(Br^{79},Br^{79}), 30%); found 425.9010 (−0.5 ppm); Anal. Calcd for $C_{12}H_{13}Br_2NO_4S$: C, 33.72; H, 3.04; N, 3.28. Found: C, 33.98; H, 2.85; N, 3.50.

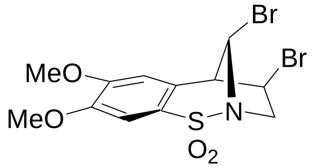

(±)-(1*R*,11*R*,12*S*)-11,12-Dibromo-4,5-dimethoxy-8-thia-9-azatricyclo[7.2.1.0^{2,7}]dodeca-2(7),3,5-triene-8,8-dioxide (18b): To a solution of alkene **5b** (75 mg, 0.28 mmol, 1 equiv) in $CHCl_3$ (10 mL) at −78 °C was added Br_2 (0.15 mL, 2.80 mmol, 10 equiv). The reaction was stirred for 15 h during which time room temperature was reached. The reaction was quenched with a saturated solution of Na_2SO_3 (20 mL) and H_2O (20mL) was added. The aqueous layer was then further extracted with CH_2Cl_2 (2 × 20 mL) and the combined organic extracts were dried over $MgSO_4$. Filtration followed by solvent removal in vacuo afforded the crude product, which was purified by flash column chromatography (c-Hex–EtOAc, 5:1) affording **18b** (104 mg, 87%) as a colourless solid. Recrystallisation from EtOAc gave crystals suitable for X-ray crystallographic analysis. mp 225 °C (EtOAc); R_f = 0.6 (c-Hex–EtOAc, 1:1); ^{1}H NMR (500 MHz, $CDCl_3$): δ 3.86 (s, 1H, 1-CH), 3.91 (s, 3H, CH_3), 3.96 (s, 3H, CH_3), 4.13 (dd, J = 5.0, 8.0 Hz, 1H, 11-CH), 4.20 (dd, J = 5.0, 15.0 Hz, 1H, 10a-CH_2), 4.60 (dd, J = 8.0, 15.0 Hz, 1H, 10b-CH_2), 6.32 (s, 1H, 12-CH), 6.70 (s, 1H, ArH), 7.19 (s, 1H, ArH); ^{13}C NMR (125 MHz, $CDCl_3$): δ 44.0 (CH), 56.3 (CH), 56.4 (CH_3), 58.5 (CH_3), 58.9 (CH), 64.5 (CH_2), 107.8 (CH), 108.5 (CH), 126.3 (C), 130.2 (C), 150.4 (C), 153.2 (C); υ_{max} (CH_2Cl_2/cm^{-1}) (NaCl) 3096, 2975, 1593, 1444, 1326, 1260, 1203, 1164, 1044; Anal. Calcd for $C_{12}H_{13}Br_2NO_4S$: C, 33.75; H, 3.07; N, 3.28. Found: C, 33.69; H, 2.99; N, 3.17.

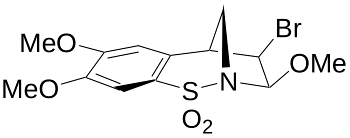
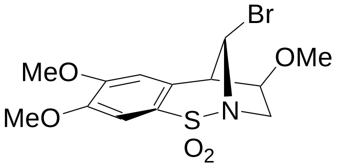

(±)-(1*R*,11*R*,12*R*)-12-Bromo-4,5,11-trimethoxy-8-thia-9-azatricyclo[7.2.1.0^{2,7}]dodeca-2(7),3,5-triene-8,8-dioxide (16b) and (±)-(1*R*,10*R*,11*R*)-11-Bromo-4,5,10-trimethoxy-8-thia-9-azatricyclo[7.2.1.0^{2,7}]dodeca-2(7),3,5-triene-8,8-dioxide (19b): To a solution of alkene **5b** (75 mg, 0.28 mmol, 1 equiv) in MeOH (5 mL) at −78 °C was added dropwise a Br_2 solution in MeOH (3 M, 0.1 mL, 0.30 mmol, 1.1 equiv). The reaction was stirred for 15 h during which time room temperature was reached. A saturated solution of Na_2SO_3 (10 mL) and H_2O (10 mL) were added and the resultant aqueous layer was further extracted with CH_2Cl_2 (2 × 10 mL). The combined organic extracts were dried over $MgSO_4$. Filtration followed by solvent removal in vacuo afforded the crude product, which was purified by flash column chromatography (c-Hex–EtOAc, 5:1) affording **16b** (61 mg, 58%) as a colourless solid. mp 210–220 °C; R_f = 0.6 (c-Hex–EtOAc, 1:1); ^{1}H NMR (400 MHz, $CDCl_3$): δ 3.46 (s, 1H, 1-CH), 3.60 (s, 3H, CH_3), 3.91 (s, 3H, CH_3), 3.94 (s, 3H, CH_3), 3.90–3.95 (m, 1H, 12a-CH_2), 4.20 (d, *J* = 13.0 Hz, 1H, 12b-CH_2), 4.51 (dd, *J* = 1.5, 5.5 Hz, 1H, 10-CH), 5.15 (d, *J* = 5.5 Hz, 1H, 11-CH), 6.71 (s, 1H, ArH), 7.17 (s, 1H, ArH); ^{13}C NMR (100 MHz, $CDCl_3$): δ 51.6 (CH), 52.4 (CH_2), 54.3 (CH), 56.3 (CH_3), 56.4 (CH_3), 57.6 (CH_3), 91.4 (CH), 107.4 (CH), 109.9 (CH), 126.7 (C), 130.1 (C), 150.1 (C), 152.7 (C); υ_{max} (CH_2Cl_2/cm^{-1}) (NaCl) 3076, 3005, 2931, 2847, 1598, 1511, 1458, 1012, 966; *m*/*z* (ESI) required 378.0013 (MH^+(Br^{79}), 100%); found 378.0011 (+0.6 ppm). Further elution gave **19b** (39 mg, 37%) as a colourless solid. mp 176–180 °C; R_f = 0.4 (c-Hex–EtOAc, 1:1); ^{1}H NMR (400 MHz, $CDCl_3$): δ 3.45 (s, 3H, CH_3), 3.68 (s, 1H, 1-CH), 3.76–3.87 (m, 2H, 11-CH, 10a-CH_2), 3.91 (s, 3H, CH_3), 3.95 (s, 3H, CH_3), 4.33 (dd, *J* = 7.0, 14.5 Hz, 1H, 10b-CH_2), 6.29 (s, 1H, 12-CH), 6.65 (s, 1H, ArH), 7.16 (s, 1H, ArH); ^{13}C NMR (100 MHz, $CDCl_3$): δ 53.3 (CH), 54.6 (CH), 56.4 (2 × CH_3), 57.6 (CH_3), 64.6 (CH_2), 85.3 (CH), 107.7 (CH), 108.4 (CH), 126.7 (C), 129.4 (C), 140.0 (C), 153.0 (C); υ_{max} (CH_2Cl_2/cm^{-1}) (NaCl) 3064, 2925, 2850, 1598, 1510, 1458, 1395, 1342, 1270, 1153, 1108, 1045, 980; *m*/*z* (ESI) required 378.0002 (MH^+(Br^{79}), 100%); found 378.0011 (−2.3 ppm); Anal. Calcd for $C_{13}H_{16}BrNO_5S$: C, 41.27; H, 4.23; N, 3.70. Found: C, 41.49; H, 4.49; N, 3.51.

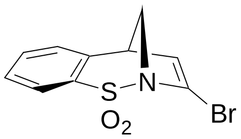

10-Bromo-8-thia-9-azatricyclo[7.2.1.0^{2,7}]dodeca-2,4,6,10-tetraene-8,8-dioxide (24a):[2] Compound **13a** (1.04 g, 2.83 mmol, 1 equiv) in THF (15 mL) was treated with a 1 M solution of TBAF in THF (20 mL, 20.00 mmol, 7 equiv) for 15 h. The solvent was removed in vacuo and CH_2Cl_2 was added (30 mL). The organic layer was washed with a saturated solution of $NaHCO_3$ (30 mL) and the product further extracted with CH_2Cl_2 (30 mL) before the combined organic layers were dried over $MgSO_4$. Filtration followed by solvent removal in vacuo afforded the crude product, which was purified by flash column chromatography (c-Hex–EtOAc, 1:1) affording **24a** (700 mg, 86%) as a colourless solid. mp 188–192 °C (CH_2Cl_2); R_f = 0.3 (c-Hex–EtOAc, 1:1); ^{1}H NMR (400 MHz, $CDCl_3$): δ 3.29 (t, *J* = 4.0 Hz, 1H, CH), 4.37 (dd, *J* = 4.0, 12.0 Hz, 1H, 12a-CH_2), 4.59 (d, *J* = 12.0 Hz, 1H, 1H, 12b-CH_2), 6.68 (d, *J* = 4.0 Hz, 1H, 11-CH), 7.12 (d, *J* = 7.5 Hz, 1H, ArH), 7.42 (t, *J* = 7.5 Hz, 1H, ArH), 7.50 (t, *J* = 7.5 Hz, 1H, ArH), 7.77 (d, *J* = 7.5 Hz, 1H, ArH); ^{13}C NMR (100 MHz, $CDCl_3$): δ 43.9 (CH), 65.5 (CH_2), 125.0 (C), 125.4 (CH), 127.4 (CH), 130.2 (CH), 132.1 (CH), 134.5 (C), 135.9 (CH), 139.3 (C); υ_{max} (CH_2Cl_2/cm^{-1}) (NaCl) 3102, 2924, 1591, 1452, 1338, 1168, 1054, 925, 866, 749; *m*/*z* (ESI) required 285.9549 (MH^+(Br^{79}), 100%); found 285.9537 (+4.1 ppm); Anal. Calcd for $C_{10}H_8BrNO_2S$: C, 41.96; H, 2.80; N, 4.90; Found: C, 41.97; H, 2.77; N, 4.71.

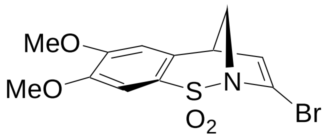

10-Bromo-4,5-dimethoxy-8-thia-9-azatricyclo[7.2.1.0^{2,7}]dodeca-2,4,6,10-tetraene-8,8-dioxide (24b): As above, compound **13b** (250 mg, 0.59 mmol, 1 equiv) was treated with 1 M solution of TBAF (10 mL, 10.00 mmol, 17 equiv) in THF (15 mL) for 15 h. The crude product was purified by flash column chromatography (c-Hex–EtOAc, 3:1) affording **24b** (137 mg, 67%) as a colourless solid. mp 178–180 °C; R_f = 0.3 (c-Hex–EtOAc, 1:1); ^{1}H NMR (500 MHz, $CDCl_3$): δ 3.20 (t, *J* = 3.5 Hz, 1H, CH), 3.90 (s, 3H, CH_3), 3.91 (s, 3H, CH_3), 4.34 (dd, *J* = 3.5, 11.5 Hz, 1H, CH_2), 4.56 (d, *J* = 11.5 Hz, 1H, CH_2), 6.58 (s, 1H, ArH), 6.70 (d, *J* = 3.5 Hz, 1H, CH), 7.12 (s, 1H, ArH); ^{13}C NMR (100 MHz, $CDCl_3$): δ 43.7 (CH), 56.3 (2 × CH_3), 65.9 (CH_2), 108.1 (CH), 109.4 (CH), 125.0 (C), 125.3 (C), 133.0 (C), 136.1 (CH), 150.0 (C), 151.5 (C); υ_{max} (CH_2Cl_2/cm^{-1}) (NaCl) 3097, 3006, 2964, 2938, 1598, 1508, 1463, 1340, 1268, 1192, 1149, 1049; *m*/*z* (ESI) required 345.9761 (MH^+(Br^{79}), 100%); found 345.9749 (+3.6 ppm).

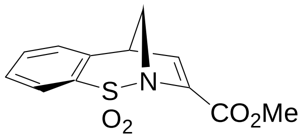

8,8-Dioxo-8λ^6-thia-9-azatricyclo[7.2.1.0^{2,7}]dodeca-2(7),3,5,10-tetraene-10-carboxylic acid methyl ester (25): Compound **24a** (300 mg, 1.05 mmol, 1 equiv) was treated with a 1.7 M solution of *t*-BuLi in pentane (1.2 mL, 2.04 mmol, 2 equiv) in a mixture of THF (5 mL) and Et_2O (5 mL) at −78 °C for 5 min. Solid CO_2 (ca. 1 g) was added and the reaction left for 15 min, warming slowly. A saturated solution of $NaHCO_3$ (15 mL) and Et_2O (10 mL) were added. The organic layer was discarded and the aqueous layer acidified with 1 M HCl solution (pH 1). The aqueous layer was then extracted with Et_2O (2 × 15 mL). The combined organic layers were dried over $MgSO_4$. Filtration followed by solvent removal in vacuo afforded the carboxylic acid intermediate, which was then treated with EDC hydrochloride (115 mg, 0.6 mmol, 0.6 equiv) and DMAP (7 mg, 0.06 mmol, 0.06 equiv) in a mixture of dry MeOH (10 mL) and CH_2Cl_2 (10 mL). The reaction was stirred at 0 °C for 5.5 h. The solvent was removed in vacuo and H_2O (20 mL) and EtOAc (10 mL) were added. The resultant aqueous layer was then further extracted with EtOAc (2 × 10 mL). The combined organic layers were dried over $MgSO_4$. Filtration followed by solvent removal in vacuo yielded the crude product which was purified by flash column chromatography (c-Hex–EtOAc, 5:1) affording the *title compound* **25** (64 mg, 23%) as a colourless solid. mp 137–142 °C (EtOAc); R_f = 0.2 (c-Hex–EtOAc, 1:1); ^{1}H NMR (500 MHz, $CDCl_3$): δ 3.50 (t, *J* = 3.5 Hz, 1H, 1-CH), 3.82 (s, 3H, CH_3), 4.27 (dd, *J* = 3.5, 12.0 Hz, 1H, 12a-CH_2), 4.59 (d, *J* = 12.0 Hz, 1H, 12b-CH_2), 7.17 (d, *J* = 7.5 Hz, 1H, ArH), 7.38 (d, *J* = 3.5 Hz, 1H, 11-CH), 7.42 (t, *J* = 7.5 Hz, 1H, ArH), 7.50 (t, *J* = 7.5 Hz, 1H, ArH), 7.71 (d, *J* = 7.5, Hz, 1H, ArH); ^{13}C NMR (100 MHz, $CDCl_3$): δ 43.3 (CH), 52.5 (CH_3), 64.5 (CH_2), 125.8 (CH), 127.5 (CH), 130.6 (CH), 132.0 (CH), 134.5 (C), 137.7 (C), 138.9 (C), 143.3 (CH), 161.2 (CO); *m*/*z* (ESI) required 266.0487 (MH^+, 100%); found 266.0487 (+0.0 ppm).

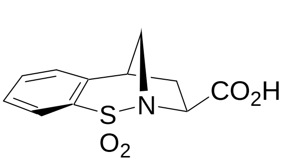

(±)-(1*S*,10*S*)-8,8-Dioxo-8λ^6-thia-9-azatricyclo[7.2.1.0^{2,7}]dodeca-2(7),3,5-triene-10-carboxylic acid (26): 10% (w/w) Pd/C (23 mg, 0.022 mmol, 10 mol%) was added to a solution of the methyl ester **25** (58 mg, 0.22 mmol, 1 equiv) in EtOH (20 mL). The mixture was degassed before stirring under a hydrogen atmosphere at room temperature for 15 h. Filtration through Celite® and solvent removal in vacuo gave the reduced methyl ester (44 mg) as a mixture of two diastereoisomers which proved inseparable by standard chromatographic techniques [*m*/*z* (ESI) required 268.0636 (MH^+, 100%); found 268.0644 (−2.8 ppm)]. Treatment of a methanolic solution (1 mL) of this

mixture with 1 M NaOMe in MeOH (5 mL, 5.00 mmol, 10 equiv) for 15 h afforded **26** (16 mg, 28%) as a colourless solid following standard acidic work-up. Crystals suitable for X-ray analysis formed on cooling to 5 °C. mp 195–197 °C; R_f = 0.55 (c-Hex–EtOAc, 1:1); ^{1}H NMR (400 MHz, $CDCl_3$): δ 2.29–2.37 (m, 1H, CH_2), 2.41–2.49 (m, 1H, CH_2), 3.32 (dd, *J* = 3.0, 6.0 Hz, 1H, CH), 3.49 (dd, *J* = 3.0, 13.0 Hz, 1H, CH_2), 4.29 (d, *J* = 13.0 Hz, 1H, CH_2), 4.62 (t, *J* = 8.0 Hz, 1H, CH), 7.20 (d, *J* = 7.0 Hz, 1H, ArH), 7.40–7.49 (m, 2H, ArH), 7.74 (d, *J* = 7.5 Hz, 1H, ArH); ^{13}C NMR (125 MHz, $CDCl_3$): δ 38.2 (CH_2), 40.2 (CH), 56.7 (CH_2), 59.4 (CH), 126.1 (CH), 127.5 (CH), 128.9 (CH), 132.7 (CH), 135.2 (C), 140.2 (C), 173.0 (CO); υ_{max} (CH_2Cl_2/cm^{-1}) 3684, 3083, 3021, 2961, 1728, 1602, 1339, 1166, 905; *m/z* (ESI) required 254.0475 (MH^+, 100%); found 254.0487 (−4.7 ppm).

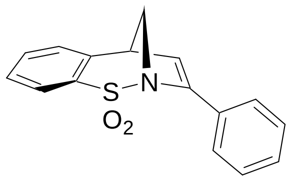

10-Phenyl-8-thia-9-azatricyclo[7.2.1.0^{2,7}]dodeca-2,4,6,10-tetraene-8,8-dioxide (27): Under N_2, a mixture of the compound **24a** (500 mg, 1.75 mmol, 1 equiv), phenylboronic acid (1.073 g, 8.80 mmol, 5 equiv), $Pd(OAc)_2$ (20 mg, 0.09 mmol, 5 mol%), PPh_3 (47 mg, 0.18 mmol, 10 mol%) and Cs_2CO_3 (1.714 g, 5.26 mmol, 3 equiv) in a mixture of THF:H_2O (10:1) (25 mL) was heated to reflux for 15 h. On cooling Et_2O (20 mL) and H_2O (20 mL) were added and the resultant aqueous layer was further extracted with Et_2O (2 × 20 mL) and the combined organic extracts were washed with a 2 M NaOH solution (20 mL) and dried over $MgSO_4$. Filtration followed by solvent removal under reduced pressure gave the crude product which was purified by flash column chromatography (c-Hex–EtOAc, 5:1) affording **27** (314 mg, 63%) as a colourless solid. mp 120–122 °C (EtOAc); R_f = 0.55 (c-Hex–EtOAc, 1:1); ^{1}H NMR (400 MHz, $CDCl_3$): δ 3.44 (t, *J* = 4.0 Hz, 1H, CH), 4.27 (dd, *J* = 4.0, 12.0 Hz, 1H, CH_2), 4.70 (d, *J* = 12.0 Hz, 1H, CH_2), 6.82 (d, *J* = 4.0 Hz, 1H, CH), 7.17 (d, *J* = 7.5 Hz, 1H, ArH), 7.31–7.39 (m, 3H, ArH), 7.40–7.47 (m, 2H, ArH), 7.69–7.71 (m, 3H, ArH); ^{13}C NMR (100 MHz, $CDCl_3$): δ 43.3 (CH), 64.3 (CH_2), 110.0 (C), 125.3 (CH), 126.6 (CH), 127.1 (CH), 127.3 (CH), 128.3 (CH), 129.2 (CH), 129.7 (CH), 131.7 (CH), 134.7 (C), 140.8 (C), 147.0 (C); υ_{max} (CH_2Cl_2/cm^{-1}) (KCl) 3063, 1965, 1592, 1452, 1327, 1162, 1029, 948; *m/z* (ESI) required 284.0733 (MH^+, 100%); found 284.0745 (−4.3 ppm); Anal. Calcd for $C_{16}H_{13}NO_2S$: C, 67.84; H, 4.59; N, 4.95. Found: C, 67.69; H, 4.65; N, 4.84.

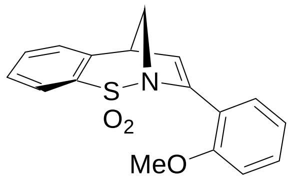

10-(2-Methoxyphenyl)-8-thia-9-azatricyclo[7.2.1.0^{2,7}]dodeca-2(7),3,5,10-tetraene-8,8-dioxide (28): According to the general procedure described above **24a** (350 mg, 1.22 mmol, 1 equiv), 2-methoxyphenylboronic acid (927 mg, 6.10 mmol, 5 equiv), $Pd(OAc)_2$ (15 mg, 0.07 mmol, 5 mol%), PPh_3 (34 mg, 0.13 mmol, 10 mol%) and Cs_2CO_3 (1.21 g, 3.71 mmol, 3 equiv) in a mixture of THF:H_2O (10:1) (20 mL) afforded **28** (289 mg, 76%) as a colourless solid. mp 162–165 °C (EtOAc); R_f = 0.6 (c-Hex–EtOAc, 1:1); ^{1}H NMR (400 MHz, $CDCl_3$): δ 3.42 (t, *J* = 4.0 Hz, 1H, CH), 3.81 (s, 3H, CH_3), 4.16 (dd, *J* = 4.0, 12.0 Hz, 1H, CH_2), 4.60 (d, *J* = 12.0 Hz, 1H, CH_2), 6.86 (d, *J* = 7.5 Hz, 1H, ArH), 7.01 (dt, *J* = 1.0, 7.5 Hz, 1H, ArH), 7.14–7.18 (m, 1H, Ar-H), 7.24 (d, *J* = 4.0 Hz, 1H, CH), 7.25–7.31 (m, 1H, ArH), 7.34–7.42 (m, 2H, ArH), 7.66–7.72 (m, 1H, ArH), 8.00 (dd, *J* = 2.0, 7.5 Hz, 1H, ArH); ^{13}C NMR (100 MHz, $CDCl_3$): δ 43.9 (CH), 55.1 (CH_3), 63.3 (CH_2), 110.4 (CH), 120.2 (CH), 120.4 (C), 125.3 (CH), 127.0 (CH), 129.4 (CH), 129.7 (CH), 130.7 (CH), 131.6 (CH), 131.8 (CH), 134.5 (C), 141.2 (C), 142.4 (C), 157.4 (C); υ_{max} (CH_2Cl_2/cm^{-1}) (KCl) 3043, 2951, 2025, 1816, 1665, 1595, 1455, 1339, 1162, 1024; *m/z* (ESI) required 314.0851 (MH^+,

100%); found 314.0855 (+1.3 ppm); Anal. Calcd for $C_{17}H_{15}NO_2S$: C, 65.18; H, 4.79; N, 4.47. Found: C, 64.48; H, 4.84; N, 4.35.

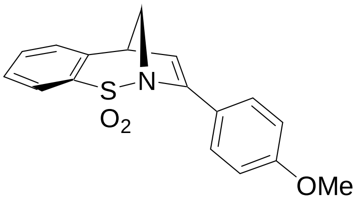

10-(4-Methoxyphenyl)-8-thia-9-azatricyclo[7.2.1.0^{2,7}]dodeca-2(7),3,5,10-tetraene-8,8-dioxide (29): According to the general procedure described a mixture of **24a** (250 mg, 0.87 mmol, 1 equiv), 4-methoxyphenylboronic acid (533 mg, 3.51 mmol, 4 equiv), $Pd(OAc)_2$ (10 mg, 0.045 mmol, 5 mol%), PPh_3 (25 mg, 0.095 mmol, 10 mol%) and Cs_2CO_3 (879 mg, 2.70 mmol, 3 equiv) in a THF:H_2O (10:1) mixture (25 mL) gave **29** (230 mg, 84%) as a colourless solid. mp 174–176 °C (EtOAc); R_f = 0.55 (c-Hex–EtOAc, 1:1); ^{1}H NMR (400 MHz, $CDCl_3$): δ 3.41 (t, *J* = 4.0 Hz, 1H, CH), 3.80 (s, 3H, CH_3), 4.24 (dd, *J* = 4.0, 12.0 Hz, 1H, CH_2), 4.68 (d, *J* = 12.0 Hz, 1H, CH_2), 6.66 (d, *J* = 4.0 Hz, 1H, CH), 6.87 (d, *J* = 9.0 Hz, 2H, ArH), 7.15 (dd, *J* = 1.5, 7.0 Hz, 1H, ArH), 7.33–7.45 (m, 2H, ArH), 7.63 (d, *J* = 9.0 Hz, 2H, ArH), 7.70 (dd, *J* = 1.5, 7.0 Hz, 1H, ArH); ^{13}C NMR (100 MHz, $CDCl_3$): δ 43.1 (CH), 55.3 (CH_3), 64.2 (CH_2), 113.8 (CH), 124.5 (C), 124.8 (CH), 125.2 (CH), 127.2 (CH), 128.0 (CH), 129.5 (CH), 131.7 (CH), 134.7 (C), 141.2 (C), 146.5 (C), 160.4 (C); υ_{max} (CH_2Cl_2/cm^{-1}) (KCl) 3070, 2947, 2841, 2045, 1606, 1508, 1454, 1329, 1251, 1171, 1026, 945; *m*/*z* (ESI) required 314.0851 (MH^+, 100%); found 314.0844 (−2.2 ppm).

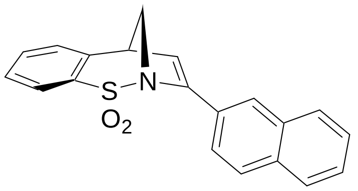

10-Naphthalen-2-yl-8-thia-9-azatricyclo[7.2.1.0^{2,7}]dodeca-2(7),3,5,10-tetraene-8,8-dioxide (30): According to the general procedure described a mixture of **24a** (250 mg, 0.87 mmol, 1 equiv) 2-naphthaleneboronic acid (602 mg, 3.50 mmol, 4 equiv), $Pd(OAc)_2$ (10 mg, 0.0045 mmol, 5 mol%), PPh_3 (24 mg, 0.09 mmol, 10 mol%) and Cs_2CO_3 (879 mg, 2.70 mmol, 3 equiv) in a mixture of THF:H_2O (10:1) (20 mL) gave **30** (242 mg, 84%) as a colourless crystalline solid. mp 204–206 °C (c-Hex–EtOAc, 5:1); R_f = 0.50 (c-Hex–EtOAc, 1:1); ^{1}H NMR (400 MHz, $CDCl_3$): δ 3.48 (t, *J* = 4.0 Hz, 1H, CH), 4.31 (dd, *J* = 4.0, 12.0 Hz, 1H, CH_2), 4.75 (d, *J* = 12.0 Hz, 1H, CH_2), 6.92 (d, *J* = 4.0 Hz, 1H, CH), 7.19 (dd, *J* = 1.5, 9.0 Hz, 1H, ArH), 7.39–7.49 (m, 4H, ArH), 7.70 (dt, *J* = 3.0, 12.0 Hz, 2H, ArH), 7.74–7.82 (m, 2H, ArH), 7.84–7.91 (m, 1H, ArH), 8.27 (s, 1H, ArH); ^{13}C NMR (100 MHz, $CDCl_3$): δ 43.3 (CH), 64.4 (CH_2), 123.5 (CH), 125.3 (CH), 126.4 (CH), 126.7 (CH), 126.9 (CH), 127.3 (CH), 127.5 (CH), 127.6 (CH), 128.0 (CH), 128.8 (CH), 129.0 (C), 129.7 (CH), 131.8 (CH), 133.1 (C), 133.6 (C), 134.7 (C), 140.8 (C), 147.0 (C); υ_{max} (CH_2Cl_2/cm^{-1}) (KCl) 3058, 2922, 2328, 1944, 1603, 1451, 1337, 1163, 1031; *m*/*z* (ESI) required 334.0902 (MH^+, 100%); found 334.0902 (+0.1 ppm); Anal. Calcd for $C_{20}H_{15}NO_2S$: C, 72.07; H, 4.50; N, 4.20. Found: C, 71.58; H, 4.60; N, 4.12.

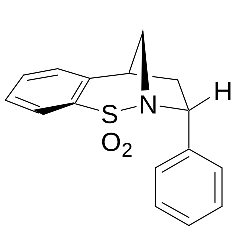

(±)-(1*S*,10*R*)-10-Phenyl-8-thia-9-azatricyclo[7.2.1.0^{2,7}]dodeca-2(7),3,5-triene-8,8-dioxide (31): 10% (w/w) Pd/C (98 mg, 0.09 mmol, 15 mol%) was added to a solution of the alkene **27** (173 mg, 0.61 mmol, 1 equiv) in DMF (20 mL). The mixture was degassed before stirring under a hydrogen atmosphere (1 atm) at room temperature for 72 h. Filtration through Celite® and solvent removal in

vacuo gave **32** (77 mg, 44%) as a colourless crystalline solid. mp 131–134 °C (EtOAc); R_f = 0.5 (c-Hex–EtOAc, 1:1); ^{1}H NMR (400 MHz, $CDCl_3$): δ 2.36 (ddd, *J* = 2.0, 7.5, 13.0 Hz, 1H, CH_2), 2.73 (ddd, *J* = 7.5, 10.0, 13.0 Hz, 1H, CH_2), 3.43 (dd, *J* = 3.5, 7.5 Hz, 1H, CH), 3.56 (dd, *J* = 3.5, 12.5 Hz, 1H, CH_2), 4.53 (dd, *J* = 2.0, 12.5 Hz, 1H, CH_2), 5.05 (dd, *J* = 7.5, 10.0 Hz, 1H, CH), 7.13–7.19 (m, 2H, ArH), 7.22–7.29 (m, 4H, ArH), 7.36 (dt, *J* = 1.0, 7.0 Hz, 1H, ArH), 7.48 (dt, *J* = 1.0, 7.5 Hz, 1H, ArH), 7.66 (d, *J* = 7.5 Hz, 1H, 1H, ArH); ^{13}C NMR (100 MHz, $CDCl_3$): δ 37.7 (CH_2), 40.1 (CH), 59.1 (CH_2), 66.4 (CH), 125.8 (CH), 127.0 (CH), 127.9 (CH), 128.2 (CH), 128.3 (CH), 129.8 (CH), 132.6 (CH), 135.1 (C), 137.2 (C), 142.1 (C); υ_{max} (CH_2Cl_2/cm^{-1}) (KCl) 3042, 2956, 2342, 1954, 1876, 1596, 1452, 1324, 1163, 1087, 975; *m*/*z* (ESI) required 284.0894 (MH^+, 100%); found 284.0902 (−2.7 ppm).

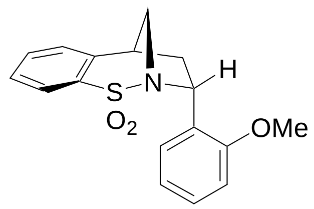

(±)-(1*R*,10*R*)-10-(2-Methoxyphenyl)-8-thia-9-azatricyclo[7.2.1.0^{2,7}]dodeca-2(7),3,5-triene-8,8-dioxide (32): As above stirring 10% (w/w) Pd/C (230 mg, 0.22 mmol, 30 mol%) and **28** (230 mg, 0.73 mmol, 1 equiv) in DMF (20 mL) under a hydrogen atmosphere afforded **32** (97 mg, 42%) as a colourless solid which was purified by flash column chromatography (c-Hex–EtOAc, 5:1). mp 198–200 °C (c-Hex–EtOAc, 5:1); R_f = 0.55 (c-Hex–EtOAc, 1:1); ^{1}H NMR (400 MHz, $CDCl_3$): δ 2.45 (ddd, *J* = 2.0, 7.0, 13.0 Hz, 1H, CH_2), 2.62 (ddd, *J* = 7.0, 10.5, 13.0 Hz, 1H, CH_2), 3.40 (dd, *J* = 3.0, 7.0 Hz, 1H, CH), 3.55 (dd, *J* = 3.0, 13.0 Hz, 1H, CH_2), 3.80 (s, 3H, CH_3), 4.56 (dd, *J* = 2.0, 13.0 Hz, 1H, CH_2), 5.38 (dd, *J* = 7.0, 10.5 Hz, 1H, CH), 6.38–6.60 (m, 2H, ArH), 6.87 (d, *J* = 8.0 Hz, 1H, ArH), 7.24–7.32 (m, 2H, ArH), 7.37 (dt, *J* = 1.0, 7.5 Hz, 1H, ArH), 7.49 (dt, *J* = 1.0, 7.5 Hz, 1H, ArH), 7.66 (d, *J* = 7.5 Hz, 1H, ArH); ^{13}C NMR (100 MHz, $CDCl_3$): δ 37.1 (CH_2), 40.1 (CH), 55.7 (CH_3), 59.0 (CH_2), 59.8 (CH), 110.7 (CH), 119.8 (CH), 123.6 (C), 125.7 (CH), 127.3 (CH), 128.2 (CH), 129.7 (CH), 129.8 (CH), 132.5 (CH), 137.7 (C), 141.9 (C), 158.7 (C); υ_{max} (CH_2Cl_2/cm^{-1}) (KCl) 3050, 2946, 1596, 1459, 1328, 1166, 1022; *m*/*z* (ESI) required 316.1007 (MH^+, 100%); found 316.0998 (−2.8 ppm).

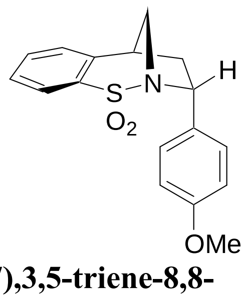

(±)-(1*S*,10*R*)-10-(4-Methoxyphenyl)-8-thia-9-azatricyclo[7.2.1.0^{2,7}]dodeca-2(7),3,5-triene-8,8-dioxide (33): As above a mixture of 10% (w/w) Pd/C (76 mg, 0.075 mmol, 15 mol%) and **29** (150 mg, 0.48 mmol, 1 equiv) in DMF (10 mL) under a hydrogen atmosphere afforded **33** (79 mg, 52%) as a colourless foam. R_f = 0.45 (c-Hex–EtOAc, 1:1); ^{1}H NMR (400 MHz, $CDCl_3$): δ 2.29 (ddd, *J* = 1.5, 7.5, 13.0 Hz, 1H, CH_2), 2.72 (ddd, *J* = 7.0, 10.0, 13.0 Hz, 1H, CH_2), 3.41 (dd, *J* = 3.0, 7.0 Hz, 1H, CH), 3.53 (dd, *J* = 3.0, 12.5 Hz, 1H, CH_2), 3.76 (s, 3H, CH_3), 4.52 (dd, *J* = 1.5, 12.5 Hz, 1H, CH_2), 5.02 (dd, *J* = 7.5, 10.0 Hz, 1H, CH), 6.77 (d, *J* = 8.5 Hz, 2H, ArH), 7.06 (d, *J* = 8.5 Hz, 2H, ArH), 7.27 (d, *J* = 7.5 Hz, 1H, ArH), 7.38 (t, *J* = 7.5 Hz, 1H, ArH), 7.49 (t, *J* = 7.5 Hz, 1H, ArH), 7.68 (d, *J* = 7.5 Hz, 1H, ArH); ^{13}C NMR (100 MHz, $CDCl_3$): δ 38.1 (CH_2), 40.2 (CH), 55.1 (CH_3), 59.1 (CH_2), 66.1 (CH), 113.3 (CH), 125.8 (CH), 127.1 (CH), 128.3 (CH), 131.0 (CH), 132.6 (CH), 137.3 (C), 142.2 (C), 159.4 (C); υ_{max} (CH_2Cl_2/cm^{-1}) (KCl) 3060, 2851, 2349, 2048, 1608, 1512, 1329, 1169, 1028; *m*/*z* (ESI) required 316.1007 (MH^+, 100%); found 316.1006 (−0.3 ppm).

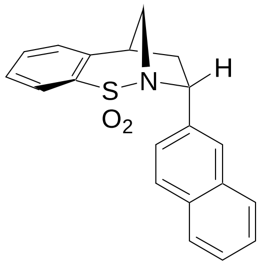

(±)-(1*S*,10*R*)-10-Naphthalen-2-yl-8-thia-9-azatricyclo[7.2.1.0^{2,7}]dodeca-2(7),3,5-triene-8,8-dioxide (34): As above a mixture of 10% (w/w) Pd/C (71 mg, 0.07 mmol, 16 mol%) and **30** (147 mg, 0.44 mmol, 1 equiv) in DMF (10 mL) were stirred under hydrogen. The crude product was purified by flash column chromatography (c-Hex–EtOAc, 5:1) affording **34** (130 mg, 88%) as a colourless crystalline solid. mp 232–234 °C (c-Hex–EtOAc, 5:1); R_f = 0.55 (c-Hex–EtOAc, 1:1); ^{1}H NMR (400 MHz, $CDCl_3$): δ 2.48 (dd, *J* = 8.0, 13.0 Hz, 1H, CH_2), 2.80 (ddd, *J* = 7.0, 9.0, 13.0 Hz, 1H, CH_2), 3.47 (dd, *J* = 3.0, 7.0 Hz, 1H, CH), 3.59 (dd, *J* = 3.0, 12.5 Hz, 1H, CH_2), 4.54 (d, *J* = 12.5 Hz, 1H, CH_2), 5.12 (app. t, *J* = 8.0, 9.0 Hz, 1H, CH), 7.30 (d, *J* = 7.5 Hz, 1H, ArH), 7.35–7.45 (m, 4H, ArH), 7.51 (t, *J* = 7.0 Hz, 1H, ArH), 7.55 (s, 1H, ArH), 7.65 (m, 2H, ArH), 7.73 (d, *J* = 8.5 Hz, 1H, ArH), 7.76 (d, *J* = 8.0 Hz, 1H, ArH); ^{13}C NMR (100 MHz, $CDCl_3$): δ 37.7 (CH_2), 40.2 (CH), 59.2 (CH_2), 66.6 (CH), 125.9 (CH), 126.0 (CH), 126.2 (CH), 127.0 (CH), 127.3 (CH), 127.6 (CH), 127.9 (CH), 128.0 (CH), 128.1 (CH), 128.4 (CH), 132.6 (CH), 132.8 (C), 132.9 (C), 133.0 (C), 137.3 (C), 142.3 (C); υ_{max} (CH_2Cl_2/cm^{-1}) (KCl) 3048, 2953, 2348, 2154, 1937, 1631, 1449, 1330, 1160, 1038; *m*/*z* (ESI) required 336.1045 (MH^+, 100%); found 336.1031 (+4.0 ppm).

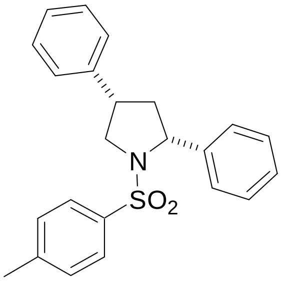

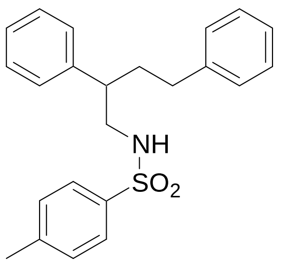

(±)-(2*R*,4*S*)-2,4-Diphenyl-1-(toluene-4-sulfonyl)pyrrolidine (35) and *N*-(2,4-diphenylbutyl)-4-methylbenzenesulfonamide (39): Under N_2 at −78 °C liquid NH_3 (ca. 100 mL) was treated with lithium wire (10 mg, 1.43 mmol, 5 equiv). This mixture was stirred for 1 h before a solution of **31** (80 mg, 0.28 mmol, 1 equiv) in THF (5 mL) was introduced in a dropwise fashion. Stirring was continued at −78 °C for 20 min before solid NH_4Cl (ca. 5 g) was added. The NH_3 was allowed to evaporate and Et_2O (25 mL) and H_2O (25 mL) were added to the residue. The resultant aqueous layer was further extracted with Et_2O (2 × 25 mL) and the combined organic extracts were dried over $MgSO_4$. Filtration, followed by solvent removal under reduced pressure afforded a colourless oil. At 0 °C the crude material was treated with Et_3N (0.06 mL, 0.43 mmol, 1.5 equiv) and TsCl (53 mg, 0.28 mmol, 1 equiv) in CH_2Cl_2 (10 mL). The mixture was stirred for 3 h during which time room temperature was reached. CH_2Cl_2 (20 mL) and H_2O (20 mL) were added and the resultant aqueous layer was further extracted with CH_2Cl_2 (2 × 20 mL). The combined organic extracts were dried over $MgSO_4$ which was removed by fitration. The solvent was removed under reduced pressure and purification of the residue by flash column chromatography (c-Hex–EtOAc, 4:1) gave initially **35** (21 mg, 20%) as a colourless oil. R_f = 0.45 (c-Hex–EtOAc, 3:1); ^{1}H NMR (400 MHz, $CDCl_3$): δ 2.05 (ddd, app. dt, *J* = 10.0, 12.5 Hz, 1H, CH_2), 2.43 (s, 3H, CH_3), 2.63–2.72 (m, 1H, CH_2), 2.90–3.02 (m, 1H, CH), 3.51 (t, *J* = 11.0 Hz, 1H, CH_2), 4.16 (ddd, *J* = 1.0, 7.0, 11.0 Hz, 1H, CH_2), 4.82 (dd, *J* = 7.0, 10.0 Hz, 1H, CH), 7.12 (d, *J* = 7.0 Hz, 2H, ArH), 7.20–7.36 (m, 10H, ArH), 7.63 (d, *J* = 8.0 Hz, 2H, ArH); ^{13}C NMR (100 MHz, $CDCl_3$): δ 21.5 (CH_3), 43.7 (CH), 44.4 (CH_2), 55.9 (CH_2), 64.5 (CH), 126.4 (CH), 127.0 (CH), 127.1 (CH), 127.3 (CH), 127.4 (CH), 128.4 (CH), 128.7 (CH), 129.6 (CH), 135.7 (C), 139.0 (C), 142.5 (C), 143.3 (C); υ_{max} (CH_2Cl_2/cm^{-1}) 3059, 2926, 1599, 1494, 1432, 1342, 1289, 1254, 1158, 1093, 1027; *m*/*z* (ESI) required 378.1535 (MH^+,

100%); found 378.1528 (+1.1 ppm). Further elution gave **39** (51 mg, 48%) as a colourless oil. R_f = 0.35 (c-Hex–EtOAc, 3:1); ^{1}H NMR (300 MHz, $CDCl_3$): δ 1.77–1.88 (m, 1H, CH_2), 1.90–2.00 (m, 1H, CH_2), 2.34–2.49 (m, 5H, CH_3, CH_2), 2.63–2.72 (m, 1H, CH), 3.00 (app. ddt, J = 4.5, 9.0 Hz, 1H, CH_2), 3.27 (app. ddt, J = 5.5, 8.0 Hz, 1H, CH_2), 4.21 (dd, J = 4.0, 4.5 Hz, 1H, NH), 7.01–7.06 (m, 4H, ArH), 7.13–7.35 (m, 8H, ArH), 7.62 (d, J = 8.0 Hz, 2H, ArH); ^{13}C NMR (100 MHz, $CDCl_3$): δ 21.5 (CH_3), 33.1 (CH_2), 35.0 (CH_2), 45.0 (CH), 48.5 (CH_2), 125.9 (CH), 127.0 (CH), 127.3 (CH), 127.8 (CH), 128.3 (CH), 128.35 (CH), 129.0 (CH), 129.6 (CH), 137.0 (C), 141.0 (C), 141.5 (C), 143.3 (C); υ_{max} (CH_2Cl_2/cm^{-1}) 3360, 2043, 2932, 1599, 1493, 1431, 1327, 1222, 1159, 1087, 810; m/z (ESI) required 380.1666 (MH^+, 100%); found 380.1684 (−4.8 ppm).

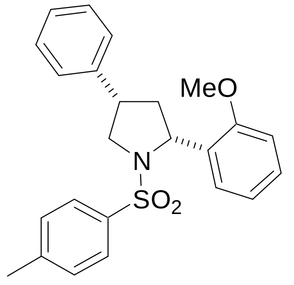

(±)-(2*R*,4*S*)-2-(2-Methoxyphenyl)-4-phenyl-1-(toluene-4-sulfonyl)pyrrolidine (36): According to the general procedure described above **32** (46 mg, 0.15 mmol, 1 equiv) in THF (5 mL) was added to a mixture of liquid NH_3 (ca. 100 mL) and lithium wire (5 mg, 0.71 mmol, 5 equiv). The pyrrolidine intermediate (46 mg) [^{1}H NMR (500 MHz, $CDCl_3$): δ 1.84–1.92 (m, 1H), 2.51–2.72 (m, 2H), 3.05–3.19 (m, 1H, NH), 3.32–3.58 (m, 2H), 3.82 (s, 3H), 4.49–4.68 (m, 1H), 6.84 (d, J = 8.0 Hz, 2H), 6.93 (t, J = 7.5 Hz, 2H), 7.13–7.24 (m, 2H, ArH), 7.26–7.30 (m, 4H, ArH), 7.47 (dd, J = 1.0, 7.5 Hz, 2H); m/z (EI) required 253.1487 (M^+, 100%); found 253.1467 (4.3 ppm)] obtained following work-up and solvent removal under reduced pressure was treated with Et_3N (0.03 mL, 0.22 mmol, 1.5 equiv) and TsCl (23 mg, 0.12 mmol, 1 equiv) in CH_2Cl_2 (10 mL). Purification by flash column chromatography (c-Hex–EtOAc, 9:1) gave **36** (15 mg, 24%) as a colourless oil. R_f = 0.25 (c-Hex–EtOAc, 3:1); ^{1}H NMR (500 MHz, $CDCl_3$): δ 2.00 (ddd, app. dt, J = 10.0, 12.5 Hz, 1H, CH_2), 2.43 (s, 3H, CH_3), 2.67–2.77 (m, 1H, CH_2), 2.82–2.97 (m, 1H, CH), 3.51 (t, J = 11.0 Hz, 1H, CH_2), 3.74 (s, 3H, CH_3), 4.12 (dd, J = 7.0, 11.0 Hz, 1H, CH_2), 5.14 (dd, J = 7.0, 10.0 Hz, 1H, CH), 6.79 (d, J = 8.0 Hz, 1H, ArH), 6.96 (t, J = 7.5 Hz, 1H, ArH), 7.11 (d, J = 7.0 Hz, 2H, ArH), 7.19–7.30 (m, 6H, ArH), 7.50 (dd, J = 1.0, 7.0 Hz, 1H, ArH), 7.60–7.67 (m, 2H, ArH); ^{13}C NMR (125 MHz, $CDCl_3$): δ 21.5 (CH_3), 41.7 (CH_2), 43.9 (CH), 55.2 (CH_3), 55.5 (CH_2), 60.2 (CH), 110.5 (CH), 120.8 (CH), 127.0 (CH), 127.1 (CH), 127.5 (CH), 127.8 (CH), 128.3 (C), 128.6 (CH), 129.5 (CH), 130.5 (C), 136.0 (C), 139.5 (C), 143.0 (C), 156.3 (C); υ_{max} (CH_2Cl_2/cm^{-1}) 3059, 2929, 1603, 1423, 1342, 1158, 905; m/z (ESI) required 408.1633 (MH^+, 100%); found 408.1617 (−4.0 ppm).

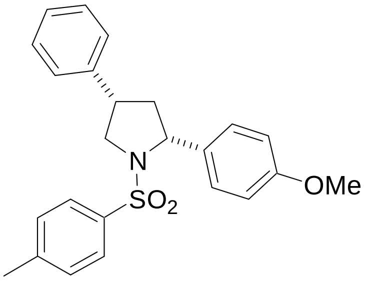

(±)-(2*R*,4*S*)-2-(4-Methoxyphenyl)-4-phenyl-1-(toluene-4-sulfonyl)pyrrolidine (37): According to the general procedure described above **33** (90 mg, 0.29 mmol, 1 equiv) in THF (5 mL) was introduced to a mixture of liquid NH_3 (ca. 100 mL) and lithium wire (10 mg, 1.43 mmol, 5 equiv). The pyrrolidine (73 mg) [^{1}H NMR (400 MHz, $CDCl_3$): δ 1.77–1.90 (m, 1H), 2.41–2.62 (m, 2H), 3.08–3.22 (m, 1H), 3.33–3.54 (m, 2H), 3.76 (s, 3H), 4.22–4.40 (m, 1H), 6.84 (d, J = 7.0 Hz, 2H), 7.17–7.23 (m, 1H), 7.27–7.33 (m, 4H), 7.32 (d, J = 7.0 Hz, 2H); υ_{max} (CH_2Cl_2/cm^{-1}) 3685, 3063, 2981, 1607, 1511, 1432, 1287, 1174, 1033; m/z (EI) required 253.1468 (M^+, 100%); found

253.1467 (+0.4 ppm)] and Et_3N (0.06 mL, 0.43 mmol, 1.5 equiv) in CH_2Cl_2 (20 mL) were treated with TsCl (54 mg, 0.28 mmol, 1 equiv) which gave **37** (47 mg, 40%) as a colourless oil following purification by flash column chromatography (c-Hex–EtOAc, 9:1). R_f = 0.45 (c-Hex–EtOAc, 3:1); ^{1}H NMR (400 MHz, $CDCl_3$): δ 2.03 (ddd, app. dt, J = 10.0, 12.5 Hz, 1H, CH_2), 2.42 (s, 3H, CH_3), 2.60–2.67 (m, 1H, CH_2), 2.91–3.01 (m, 1H, CH), 3.49 (t, J = 11.0 Hz, 1H, CH_2), 3.79 (s, 3H, CH_3), 4.14 (ddd, J = 1.0, 7.5, 11.0 Hz, 1H, CH_2), 4.76 (dd, J = 7.0, 10.0 Hz, 1H, CH_2), 6.82 (d, J = 8.5 Hz, 2H, ArH), 7.13 (d, J = 7.0 Hz, 2H, 2H, ArH), 7.20–7.28 (m, 7H, ArH), 7.61 (d, J = 8.5 Hz, 2H, ArH); ^{13}C NMR (100 MHz, $CDCl_3$): δ 21.5 (CH_3), 43.5 (CH), 44.3 (CH_2), 55.3 (CH_3), 55.8 (CH_2), 64.0 (CH), 113.8 (CH), 127.0 (CH), 127.1 (CH), 127.4 (CH), 127.7 (CH), 128.6 (CH), 129.5 (CH), 134.5 (C), 135.8 (C), 139.2 (C), 143.2 (C), 158.9 (C); υ_{max} (CH_2Cl_2/cm^{-1}) 3059, 2966, 1607, 1511, 1431, 1343, 1305, 1253, 1159, 1096, 1032; *m*/*z* (ESI) required 408.1633 (MH^+, 100%); found 408.1626 (−1.8 ppm).

References

1. Evans, P.; McCabe, T.; Morgan, B. S.; Reau, S. *Org. Lett.* **2005,** *7,* 43–46. doi:10.1021/ol0480123
2. Dura, R. D.; Paquette, L. A. *J. Org. Chem.* **2006,** *71,* 2456–2459. doi:10.1021/jo0526587
